# Interethnic Interaction, Strategic Bargaining Power, and the Dynamics of Cultural Norms: A Field Study in an Amazonian Population

**DOI:** 10.1007/s12110-017-9297-8

**Published:** 2017-08-18

**Authors:** John Andrew Bunce, Richard McElreath

**Affiliations:** 1 Department of Human Behavior, Ecology, and Culture, Max Planck Institute for Evolutionary Anthropology, Leipzig, Germany; 2 Department of Anthropology, University of California, Davis, CA USA; 3 Department of Anthropology, Indiana University, Bloomington, IN USA

**Keywords:** Ethnic boundaries, Cultural evolution, Education, Norms, Indigenous peoples, Amazonia

## Abstract

Ethnic groups are universal and unique to human societies. Such groups sometimes have norms of behavior that are adaptively linked to their social and ecological circumstances, and ethnic boundaries may function to protect that variation from erosion by interethnic interaction. However, such interaction is often frequent and voluntary, suggesting that individuals may be able to strategically reduce its costs, allowing adaptive cultural variation to persist in spite of interaction with out-groups with different norms. We examine five mechanisms influencing the dynamics of ethnically distinct cultural norms, each focused on strategic individual-level choices in interethnic interaction: bargaining, interaction-frequency-biased norm adoption, assortment on norms, success-biased interethnic social learning, and childhood socialization. We use Bayesian item response models to analyze patterns of norm variation and interethnic interaction in an ethnically structured Amazonian population. We show that, among indigenous Matsigenka, interethnic education with colonial Mestizos is more strongly associated with Mestizo-typical norms than even extensive interethnic experience in commerce and wage labor is. Using ethnographic observations, we show that all five of the proposed mechanisms of norm adoption may contribute to this effect. However, of these mechanisms, we argue that changes in relative bargaining power are particularly important for ethnic minorities wishing to preserve distinctive norms while engaging in interethnic interaction in domains such as education. If this mechanism proves applicable in a range of other ethnographic contexts, it would constitute one cogent explanation for when and why ethnically structured cultural variation can either persist or erode given frequent, and often mutually beneficial, interethnic interaction.

**Electronic supplementary material:**

The online version of this article (doi:10.1007/s12110-017-9297-8) contains supplementary material, which is available to authorized users.

The extent of human cultural variation is unprecedented in nature, and its evolutionary origins remain mysterious. Much human cultural variation is structured in symbolically marked groups (*ethnic groups*), which are often associated with different suites of beliefs about what constitutes appropriate behavior in a given context—cultural norms (Barth 1998; see also Bicchieri 2006). Life in such groups may have characterized our species for 80,000 years or more (d'Errico et al. 2009; Foley and Lahr 2011). The facts that ethnic groups are nearly universal in human populations at all but the smallest geographic scales, are absent in all other primates, and are highly dynamic present important puzzles to evolutionary social scientists: What are the psychological and cultural processes that make and remake ethnic groups? What role did such processes play in the adaptive history of humankind?

## Evolutionary Models of the Norms and Behaviors of Symbolically Marked Groups

One idea to explain the maintenance of ethnic groups is that assortment on symbolic markers helps to preserve locally adaptive norms, knowledge, and beliefs from erosion by admixture (Henrich and McElreath 2003; Richerson and Boyd 2005). For example, McElreath et al. (2003) demonstrate that symbolically marked groups with differences in norms can arise spontaneously under a minimal set of assumptions: benefits to interindividual interaction depend on norm coordination, interaction partners are chosen on the basis of markers, and norms and markers tend to be acquired from locally successful individuals. Once such marked groups evolve, intragroup coordination interactions yield greater benefits than interaction between groups. A similar model by Boyd and Richerson (1987) focuses instead on ecological adaptations rather than norms, but it also produces symbolically marked groups that protect adaptive, culturally transmitted behavioral variation from erosion by mixing between residential groups. Once evolved, ethnic groups in these models have salient, and relatively fixed, boundaries (see also Pagel and Mace 2004), and isolating mechanisms attenuate costly intergroup interaction (analogous to reproductive isolating mechanisms in biological species; e.g., Price 2008).

A shortcoming of these models is that interaction between groups is exogenously imposed, rather than a result of the strategic decisions of individuals. In these models, there are no benefits to intergroup over intragroup interaction. In contrast, in nearly all ethnographic and historically described contexts, people voluntarily engage in at least some interethnic interaction (e.g., Wolf 1982). Such interaction sometimes entails the adoption of out-group cultural norms (Behrens 1992; Kopenawa and Albert 2013; Nagel 1996) and is not necessarily viewed by participants as detrimental (Baer 2004; Rosengren 2004). There may be adaptive reasons to interact with other ethnic groups, and even to adopt culturally transmitted behavior from them. Thus, models that exclude such benefits miss a potentially important factor affecting cultural dynamics.

One potential resolution to this conflict between models and empirical observation is that individuals strategically adjust how, and with whom, they interact, in order either to reduce costs of norm miscoordination or, rather, to preferentially adopt adaptive behavior from members of other groups. The strategic nature of between-group interaction could help explain how, in some contexts, ethnic-typical norms can be maintained for generations despite frequent interethnic interaction (e.g., the Fur and Baggara of western Sudan: Haaland 1998), while, in other cases, the norms of one ethnic group are rapidly replaced by those of another (e.g., assimilation of some immigrants in the United States: Gans 1979). The lack of mechanistic models of these dynamics represents an important challenge for our understanding of the evolution of cultural norms in ethnically structured populations. Ultimately we seek a body of theory that can explain the uniqueness of human ethnic variation among primates, contribute to an understanding of the dynamics of historical and contemporary ethnic groups, and enhance the ability of ethnic minority members to reverse or slow the loss of valued cultural norms, should they wish to.

## Mechanisms of Norm Adoption at Ethnic Boundaries

Previous research on ethnic variation has emphasized links between the adoption of out-group cultural norms (e.g., assimilation and marginalization), participation in interethnic interaction (e.g., through social networks), individual personality traits (e.g., attitude toward the out-group, bicultural efficacy), and the strategic maintenance or change of ethnic identity (Berry 1997; LaFromboise et al. 1993; Wimmer 2013). However, the mechanistic links between interethnic interaction and norm adoption are often not specified in sufficient detail to facilitate empirical testing. For instance, the pioneering study of Graves (1967) among minority Spanish-speaking Americans and Native Americans in a majority Anglo-American town in the southwestern US, found a strong relationship between interethnic interaction (e.g., in domains such as education, military service, out-group friendships, employment) and the probability that minority individuals hold majority-typical norms. From this, it is argued that exposure to out-group norms, identification with the out-group, and access to resources controlled by the out-group all play roles in the adoption of out-group norms. However, the mechanisms underlying such relationships are not well understood, making it difficult to distinguish among them with the available data. As an initial step forward, we focus on five theoretically grounded and previously published mechanisms whose predictions can be compared using quantitative and ethnographic data at hand. An individual’s adoption of out-group norms may depend on: (1) differential bargaining power during interethnic interactions, (2) the relative importance (e.g., relative frequency) of interethnic coordination interactions; (3) interethnic assortment on coordination norms, coupled with success-biased social learning among co-ethnics; (4) success-biased interethnic social learning; and (5) favorable exposure to out-group norms during childhood socialization. These five mechanisms are neither exhaustive nor mutually exclusive. However, they make distinct predictions that we can evaluate using empirical data in order to better understand their roles in the norm dynamics of a particular ethnically structured population. In the remainder of this section, we sketch each and provide citations to further discussion.

### Bargaining

Norms play an important role in interethnic interactions because many such interactions have the form of coordination games. In a coordination game, all participants receive a higher (though not necessarily equal) payoff if they act in concordant rather than discordant manners (Bicchieri 2006). Consequently, players should seek to interact with others holding similar norms in the context of interaction (McElreath et al. 2003). Many domains of social life, such as commerce, healthcare, education, and marriage (Nave 2000), can be modeled as coordination games. Interethnic interactions can be especially challenging because, initially, distributions of norms often differ among ethnic groups (Barth 1998), potentially frustrating attempts at coordination (e.g., eighteenth-century Chinese-British commerce: Sahlins 1994).

When two individuals with different norms desire to coordinate, they must negotiate about whose norm they will use for the interaction. In many contexts of social coordination, the relevant norms may be deeply held and psychologically costly to change—for example, norms of fairness, child-rearing, and education. Individuals may be able to coordinate using norms different from those with which they personally identify (e.g., cross-cultural or bicultural competence: Johnson et al. 2006; LaFromboise et al. 1993), but the resulting psychological costs (e.g., cognitive dissonance: Festinger 1962), among other potential costs, should motivate them to try to impose their preferred norm on the coordination interaction. Following Ensminger and Knight (1997; Knight 1992), we define an individual’s ability to do this as *bargaining power*. Bargaining power is contingent on, among other things, control over the resources (material or immaterial) that one brings to the coordination interaction, as well as control over choice of interaction partner. An individual’s ability to impose coordination norms is reduced if she has less control over (or less-valuable) coordination resources and if she has less choice about whom she coordinates with (Ensminger and Knight 1997; Knight 1992).

When there are benefits to interethnic coordination but limited numbers of out-group coordination partners, members of one ethnic group (e.g., residents) may compete among themselves to interact with members of the other ethnic group (e.g., visitors). One way that residents can compete for visitors is to offer to coordinate using visitor-typical, rather than resident-typical, norms. Thus, in a given coordination interaction, a visitor has greater bargaining power than a resident, all else being equal, because she can choose from among residents the one most willing to coordinate using her preferred norm. In the terminology of economics, the visitor has short-side power in a contested exchange (Bowles and Gintis 1993). In this way, where there is interethnic asymmetry in resource control, intraethnic competition among members of the resource-favored group tends to decrease any interethnic asymmetry in bargaining power (Ensminger and Knight 1997). The effect of differential bargaining power on norm adoption is mediated by the frequency and importance of interethnic interactions. The more an individual engages in subjectively important interactions using particular norms, the more likely she may be to adopt and internalize those norms, changing her cognition to align with her behavior (Barth 1966; Festinger 1962). Thus, we predict that the probability that an individual adopts out-group norms, as opposed to maintaining her original norms, is a decreasing function of the relative amount of bargaining power she has in frequent and important interethnic coordination interactions.

### Interaction-Frequency-Biased Norm Adoption

Regardless of relative bargaining power, the norms of frequently interacting ethnic groups would be expected to evolve toward similarity in any domain of interethnic interaction (Barth 1998) since both parties would then receive the benefits of coordination (e.g., market-based interactions: Henrich et al. 2010). We predict that, in a given interaction context, individuals on one side of the interaction should directly adopt the norm of their most frequent or most important coordination partners, all else being equal. For instance, given a degree of asymmetry in bargaining power across a range of interaction contexts, norm adoption by low-power individuals will be most likely in those domains where interaction is most frequent or most important to them. If interethnic interactions are important, this mechanism will produce a positive association between out-group norms and interethnic interaction experience on one side of the ethnic boundary.

### Interethnic Assortment on Norms

If there is preexisting intragroup variation in norms, individuals may preferentially assort such that those with the most out-group-typical norms engage in the most interethnic coordination. If there is an added benefit to interethnic over intraethnic coordination, individuals who have out-group-typical norms in the context of interaction may attain high prestige. Out-group norms can then spread within a group as people copy prestigious or successful co-ethnics (Barth 1966; Henrich and Gil-White 2001), with the possible result that the norms of one group eventually replace those of the other. This process can result in a positive association between the amount of interethnic interaction and the probability of holding out-group-typical norms, an empirical pattern potentially indistinguishable from that generated by the previous mechanism of interaction-frequency-biased norm adoption. However, if interethnic assortment on norms is operating, we expect the probability that an individual initially engages in interethnic interaction to be higher if she already holds out-group-typical norms. Without assortment, initial interethnic interaction is expected to be random with respect to norms.

### Success-Biased Interethnic Social Learning

An individual’s decision to adopt the norms of another ethnic group may be based on the relative perceived success or prestige of individuals in the other ethnic group relative to that of those in her own group. When there is a causal link between a socially learned norm and group-level success (e.g., when group benefits depend, in a non-additive manner, on the frequency of the norm among its constituents), the selective imitation of successful groups can be operationalized as cultural group selection (Henrich 2004; Richerson et al. 2016). This may occur with or without direct interaction between individuals in the two groups, as long as the norms of the successful or prestigious group are known to individuals in the other. Thus, success-biased interethnic social learning of norms need not depend on the benefits or costs to individuals of interethnic coordination interactions. Boyd and Richerson (2002) showed that, under a range of conditions, this mechanism can result in the spread of individually costly group-beneficial norms among ethnic groups, and it may explain the spread of early Christian norms of mutual aid (reviewed in Richerson and Boyd 2005). However, such success-biased social learning is easily generalized to any norm that people wish to copy, whether or not the norm itself entails group benefits. One type of evidence for this mechanism would come from an observation that the norms typical of a successful or prestigious ethnic group are directly copied by individuals in other ethnic groups, regardless of those individuals’ personal interethnic interaction experience in the social contexts in which such norms apply. Note that the principal difference between this mechanism and the previous mechanism of interethnic assortment on norms is that, here, the targets of success-biased social learning tend to be individuals in the out-group rather than co-ethnics with out-group-typical norms.

### Childhood Socialization

Socialization is the process by which children are taught the norms of the society or ethnic group in which they live. Although some norms may correspond to a near-universal genetic predisposition (e.g., mating aversion for very close kin: van den Berghe 1983), it is likely that, on average, children are not born with a strong predisposition one way or the other toward most norms responsible for ethnic variation in our species. The cultural pluripotency of young children is most apparent in situations of cross-cultural fostering, where children readily adopt norms very different from those of their biological parents (reviewed in Richerson and Boyd 2005), and in societies undergoing rapid social and economic change—for instance, where norms covary with birth cohort (reviewed in Chen and French 2008). Children have evolved high receptivity to learning whichever norms are used to socialize them (Legare and Nielsen 2015) since this facilitates access to the benefits associated with integration into the society where they expect to grow up (Chen and French 2008; Ochs and Izquierdo 2009).

The purpose of norms, by definition, is to restrict the range of available behavioral choices in a given decision context, at times eliminating choices that might otherwise seem desirable (e.g., a norm against cheating). In order to function, a norm must be reasonably inflexible given contextual variation in the behavior to which it applies (e.g., cheating on an exam versus cheating in sports). However, this same quality of inflexibility may make norms, once learned, relatively resistant to change later in life, even when there may be benefits to doing so (e.g., norms of circumcision learned prior to immigration into a non-circumcising host society: Morison et al. 2004). For this reason, a child socialized using an in-group norm may become progressively more resistant to subsequent adoption of a conflicting out-group norm. It may also be the case that children pass through a developmental window (perhaps prior to adolescence) in which they are cognitively more receptive to the adoption of cultural norms (Cheung et al. 2011; but see Chudek et al. 2015). If either norm inflexibility or a developmental norm-adoption window occur (or if both occur), we predict that, on average over a broad range of ages, the earlier in the socialization process (e.g., the younger) an individual is favorably exposed to out-group norms through interethnic interaction, the more likely she is to adopt norms of the other ethnic group.

## Overview of the Present Study

Incorporating the mechanisms described above, we develop and evaluate hypotheses for why interethnic interaction variably results in persistence or erosion of cultural norm differences. We do so using ethnographic data on interethnic interaction and norm adoption decisions in a relatively remote population in Amazonian Peru, where a salient ethnic boundary exists between indigenous Matsigenka and neighboring colonist Mestizos. Matsigenka live in a community legally protected from incursion by Mestizos, and with very little exposure to mass media. Matsigenka-Mestizo interaction is almost entirely constrained to the domains of commerce, wage labor, and education, and such interethnic interaction nearly always requires Matsigenka to leave their community and travel to Mestizo towns. Hence, the circumstances of interethnic interaction are highly asymmetric and limited to specific memorable contexts. This allows us to abstract away from the full complexity of interethnic frontiers and collect individual-level data on both norms and histories of interethnic interaction that address hypothetical mechanisms for the persistence or erosion of norm differences.

We find that Matsigenka who interact voluntarily, and even frequently, with Mestizos in the contexts of labor and commerce show little evidence of change towards Mestizo-typical norms, whereas those who attended Mestizo schools are more likely to hold Mestizo-typical norms as adults. A number of mechanisms likely contribute to this empirical pattern. However, using ethnographic data we argue that, in particular, the differential bargaining power of each ethnic group in each domain of interaction provides both a theoretically cogent and an empirically accurate explanation that extends existing evolutionary models of the cultural dynamics of ethnic groups in human societies. Furthermore, we suggest that changes in bargaining power in the context of intercultural education may contribute to the maintenance of cultural norms valued by ethnic minorities.

This paper employs multilevel Bayesian item-response models to analyze patterns of individual-level variation in cultural norms. This framework is consistent with, and meaningfully advances, quantitative anthropological approaches to norm variation (e.g., cultural consensus and consonance: Dressler et al. 2005; Oravecz et al. 2014, 2015; Romney et al. 1986). These models treat norm variation as directly unobservable, but nevertheless inferable from patterns of behavior (e.g., responses to interview questions), and make no assumptions about the correctness or incorrectness of any norm or behavior. We provide a script sufficient to repeat and extend our analysis so that other researchers can engage with these models and adapt them to other contexts.

## Methods

### Study Population

**Fig. 1:**
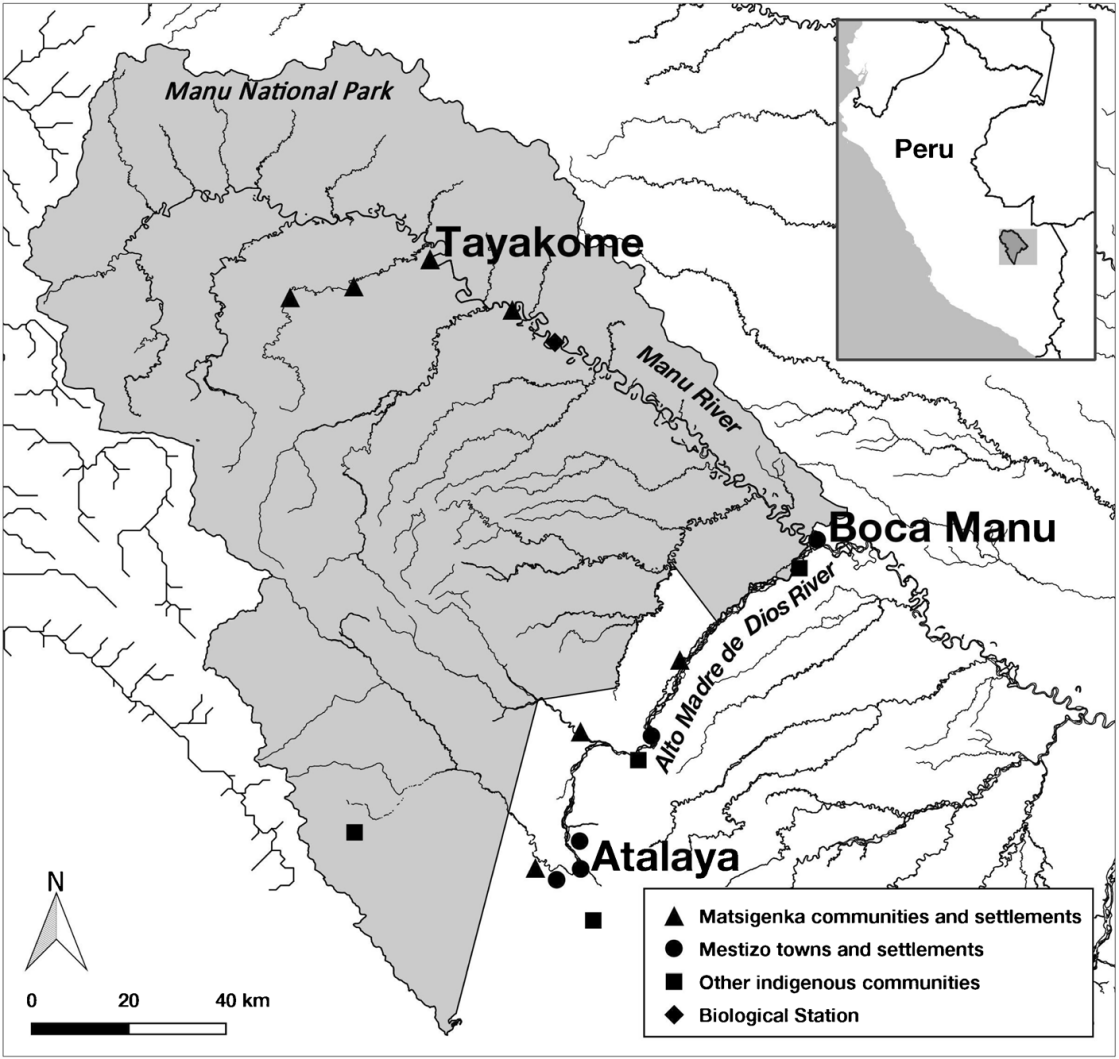
Map of the Matsigenka study community of Tayakome, and the Mestizo study communities of Boca Manu and Atalaya, as well as the locations of other nearby settlements in and around Manu National Park (*in gray*), Peru

The study was conducted among residents of the Matsigenka native community of Tayakome (adult population: 79), located inside Manu National Park, in the department of Madre de Dios, in the lowland Amazonian region of southeastern Peru, and in the Mestizo towns of Boca Manu (adult population: ~80) and Atalaya (adult population: ~65), located just outside the boundary of the park, in the departments of Madre de Dios and Cusco, respectively (Fig. 1).

Tayakome residents practice swidden horticulture, fishing, and hunting. Most Matsigenka understand basic Spanish but prefer to interact in their own language. Some travel outside the park and live among Mestizos for weeks to months working as wage-laborers (32%), or to attend Mestizo-run schools (20%). Shorter excursions are occasionally made to purchase items (e.g., pots, flashlights) in Mestizo general stores. There is a government health post in Tayakome, though most adult Matsigenka interact little with the Mestizo technician. The primary school in Tayakome is staffed by externally trained Matsigenka teachers. Nearly all interethnic interaction with Mestizos occurs in the three domains of commerce, wage labor, and education, and takes place in Mestizo towns. There is very little Matsigenka-Mestizo intermarriage or domestic interaction. Most Mestizos are colonists from the Andean highlands who usually interact with each other in Spanish. They are not permitted to enter the national park without a government permit. Residents of Boca Manu and Atalaya tend small general stores and restaurants, build wooden boats, grow plantains for the market, log the surrounding forest, and/or work in tourism for the park. Further details of the study communities can be found in ESM A.1, as well as in Shepard et al. (2010) and Llosa Isenrich and Nieto Degregori (2003).

### Data Collection

JAB lived in Boca Manu for approximately three months (September and November 2012, January 2014), Atalaya for two months (December 2012 and February 2014), and Tayakome for 13 months (January–December 2013, March 2014). Over several rounds of semi-structured interviews, he recorded interviewees’ life history and recollections of personal intra- and interethnic interaction experience, emphasizing the domains of commerce, wage labor, and education. He then designed a set of fourteen vignette questions (Table 1) for the purpose of learning about specific norms in eight contexts of social coordination (commerce, wage labor, education, spousal relations, parent-offspring relations, inheritance, healthcare, and religion) and administered these questions privately to 74 (94%) residents of Tayakome (including the Mestizo health technician), 45 (56%) residents of Boca Manu, and 42 (65%) residents of Atalaya, all of whom had been previously interviewed regarding life history and interethnic interaction experience. Further description of data collection methodology, as well as translations of vignette questions are provided in ESM A.2.

**Table 1:** Vignette questions administered in this study, and their respective social contexts. The response corresponding to a meta-norm for practical interdependence is indicated in column four. An alternative response corresponds to a meta-norm for respectful autonomy. The number of Matsigenka and Mestizo interviewees answering each question is indicated in column five. A parenthetical “A” indicates that the question was only asked of Mestizos in Atalaya, not Boca Manu. Further explanation and translations of these questions are provided in ESM A.2

Number	Social context	Question	Practical interdependence	*N* (Matsi, Mest)
1	Spousal Relations	There is a married couple with no children. The woman hunts and fishes (Mestizo: has a job and makes more money). The man stays home and cooks, weaves (Mestizo: cleans), and washes clothes. Is this okay or not okay?	Okay	71, 80
2	Parent-Offspring Relations	After school, a ten-year-old daughter cannot go to a friend’s house to play because she has to care for her two-year-old brother until their parents come home at night. Is this okay?	Okay	77, 78
3	Inheritance	A man always wears his favorite hat. After he dies, his son takes the hat and wears it. When he wears it he remembers his father. Is this okay?	Not okay	74, 79
4	Education	A teacher hits students when they don’t learn. Is this okay?	Okay	75, 74
5	Education	A student pays attention to the teacher and never asks any questions. Is this okay?	Okay	79, 81
6	Healthcare	If you get a respiratory illness (influenza), do you first go to the health post, first use home remedies, or first go to a shaman or curandero?	Health post	79, 81
7	Inheritance	An old woman has two new pots and two adult daughters. One daughter has her own two pots, but wants her mother’s pots. The other daughter has no pots, and also wants her mother’s pots. When the mother dies, who should inherit the pots? Illustrated with a diagram. Options: one pot to each daughter, both pots to the daughter who has none.	Both pots to the daughter who has none	76, 82
8	Religion	A good person does not want to be baptized. Where does his or her soul go when they die? Options: up (heaven), down (hell), somewhere else.	Hell or somewhere else	64, 60
9	Religion	A bad person is baptized. Where does her or his soul go when they die? Options: up (heaven), down (hell), somewhere else.	Heaven	64, 29 (A)
10	Wage Labor	A man is hired to prepare an agricultural field. He stops work at noon in order to go visit a friend. He returns the next day to finish the job. Is this okay?	Okay	75, 77
11	Commerce	There are two stores. One is cheap with a mean owner. The other is expensive with a nice owner. Where would you buy?	Cheap store with mean owner	63, 77
12	Spousal Relations	A man wants to marry. His mother is the sister of the woman’s father. Is it okay for him to marry this woman? Illustrated with a diagram. For the Matsigenka, examples were provided of potential marriages between people known to the interviewee.	Okay	70, 30 (A)
13	Parent-Offspring Relations	Parents want their daughter to marry a certain man that she does not like. She wants to marry someone else. Should she obey her parents and marry him anyway or not?	Obey parents	63, 81
14	Wage Labor	A man is hired to work two days: Monday and Tuesday. Monday night there is a party (Matsigenka: hosted by a Matsigenka). Should he go and get drunk? (Matsigenka: He goes and gets so drunk that he can’t work on Tuesday. Is this ok?)	Okay to go and become drunk^a^	67, 80

^a^See ESM A.2 for an explanation of the cultural context of drunkenness in Matsigenka society, which will differ from that of most readers

### Statistical Analysis

The norms measured by the vignette questions may covary, such that knowing how an individual answered one question gives you information about how she answered another question, and, in the ideal case, about how she answered all of the other questions. If true, then people’s responses to the fourteen vignettes can be represented by a smaller number of latent dimensions, and, ideally, by a single latent dimension. We use Item Response Theory (IRT) models (Bafumi et al. 2005; Fox 2010; Jackman 2001; Schacht and Grote 2015) in a Bayesian framework (McElreath 2016) to show that, for this study, the vignette responses of each interviewee are well represented by a single dimension. This latent dimension constitutes a convenient way to compare individuals on the basis of all fourteen measured norms simultaneously. It does not necessarily represent a unitary, overarching belief held by actual people (e.g., a meta-norm). For instance, it may be that the fourteen measured norms are functionally independent but happen to covary within this sample of people. However, for ease of exposition below, we will refer to the latent dimension as representing a meta-norm. We interpret the negative pole of the dimensional axis as a meta-norm prioritizing respectful autonomy, while the positive pole represents a meta-norm prioritizing practical interdependence. See ESM A.2 for the relation between individual vignette questions and interpretation of the latent dimension. Note, however, that the conclusions below do not depend on interpretation of this constructed dimension. The location on the latent axis of individual *j* (and thus a continuous measure of *j*’s meta-norm) is represented by the parameter α_*j*_. We model α_*j*_ as a linear function of a population-mean intercept (*b*_0_), an individual-level random effect (*b*_indiv[*j*]_, zero-centered offset from *b*_0_ for each individual *j*), and various combinations of hypothesized predictors, including ethnicity, and interethnic commerce, wage labor, and education experience:

$$ {\upalpha}_j={b}_0+{b}_{\mathrm{indiv}\left[j\right]}+{b}_1{x}_{1\left[j\right]}\dots, \mathrm{for}j=1,\dots, J $$

where *J* is the number of interviewees. An example fixed effect predictor, *b*_1_ *x*_1[*j*]_, is the product of the coefficient for ethnicity and the binary ethnicity indicator for individual *j*.

To construct an IRT model, we follow Bafumi et al. (2005) by embedding the linear function α_*j*_ within a logistic function. This allows us to simultaneously evaluate properties of each individual and each vignette question with respect to the latent dimension. The probability that the response *y* of a particular interviewee *j* to a particular vignette question *k* is the practical interdependence response (column four of Table 1), Pr(*y*_*jk*_ = interdependence), is given by a logistic function (inverse logit) ranging between zero and one:

$$ \Pr \left({y}_{jk}=1\right)={\mathrm{logit}}^{-1}\left[{\gamma}_k\left({\upalpha}_j-{\upbeta}_k\right)\right] $$

where practical interdependence and respectful autonomy responses are represented by 1 and 0, respectively. The domain (*x* axis) of this logistic function is the latent dimension. The slope at the function’s inflection point, γ_*k*_, is the degree to which an affirmative versus negative response to question *k* discriminates among individuals holding the meta-norm for respectful autonomy versus practical interdependence. The location of the inflection point on the latent axis, β_*k*_, is the degree to which a person must hold the meta-norm for practical interdependence in order for the model to predict that she give the interdependence-associated response to question *k*. See ESM Figure B1 for illustrations.

To check the robustness of results to the effects of different predictors, we fit a series of 19 models varying in the fixed effect predictors included in the linear function for α_*j*_. Parameter estimation for each model was accomplished with RStan (Stan Development Team 2016), running four Hamiltonian Monte Carlo chains in parallel until convergence was suggested by a high effective number of samples (>500) and $\hat{R}$ estimates of 1.00 (McElreath 2016). This entailed 4000 samples per chain, half of which were warm-up. We compared model fit with WAIC (McElreath 2016). Data and statistical analysis scripts in R (R Core Team 2014) implementing RStan are available from Github (https://github.com/jabunce/bunce-mcelreath-HN-2016-matsigenka-norms). Further explanation and priors for model parameters are provided in ESM A.3.

## Results

### Associations between Norms and Interethnic Experience

**Fig. 2:**
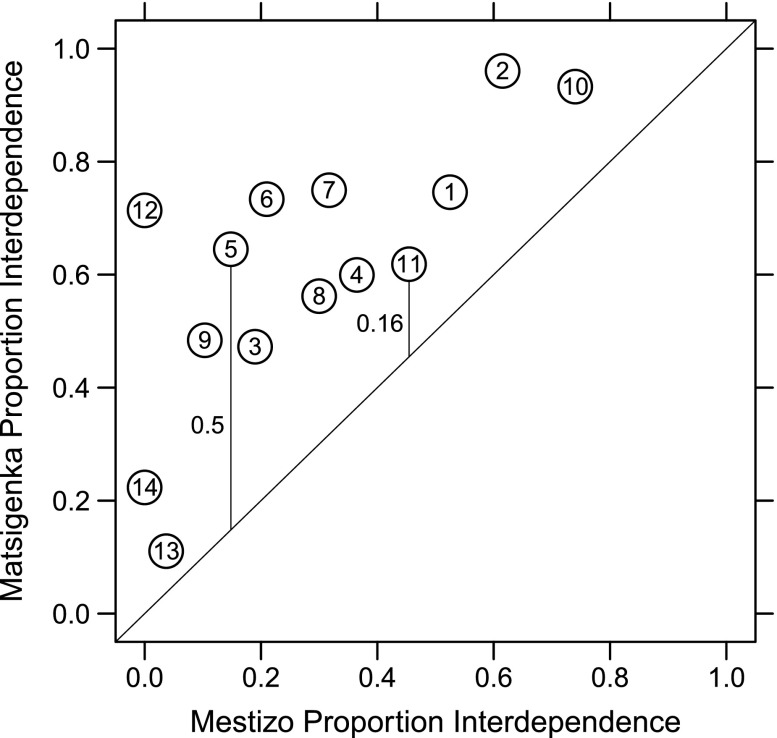
Proportions of Matsigenka (*n* = 79) and Mestizo (*n* = 82) interviewees giving the practical interdependence response to the fourteen vignette questions in Table 1. The diagonal is the line of equal proportions between Matsigenka and Mestizos. The vertical (or horizontal) distance from a point to the diagonal is the difference in proportion between ethnic groups. Example differences for questions 5 and 11 are given. Note that, for all questions, a larger proportion of Matsigenka than Mestizos gave interdependence responses, i.e., all points fall above the diagonal

Figure 2 shows that, for all fourteen vignette questions, a larger proportion of Matsigenka than Mestizos gave responses corresponding to practical interdependence. These raw proportions suggest an overall ethnic difference in the distributions of the norms applied by the interviewees to answer the questions, and they demonstrate the utility of the interview instrument to distinguish between these two ethnic groups. See ESM C.1 for further discussion of this result. In all nineteen IRT models, posterior estimates of the discrimination parameters (γ_*k*_) for all vignette questions are non-zero (ESM Figures B1 and B2). This indicates that all questions can reasonably distinguish among individuals along a single latent axis (Jackman 2001), which, as described above, we interpret as representing an individual’s meta-norm for practical interdependence versus respectful autonomy.

**Fig. 3:**
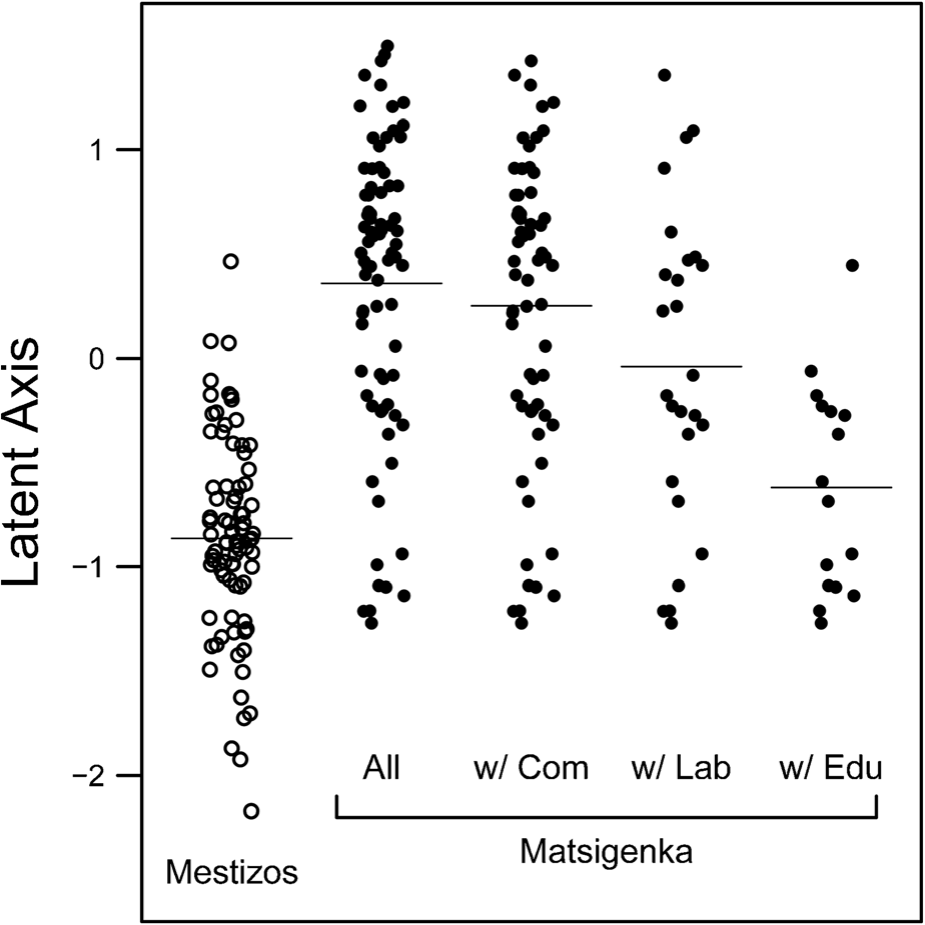
Mean posterior probability estimates for the locations (α_*j*_) of all 161 interviewees on the latent axis, as predicted by an IRT model with a random effect for individual and no predictors (model m1 in ESM Table B1). Plotted in columns from left to right are: all Mestizos, all Matsigenka, only Matsigenka who have commerce experience with Mestizos (w/ Com), only Matsigenka who have wage labor experience with Mestizos (w/ Lab), and only Matsigenka who have education experience with Mestizos (w/ Edu). Horizontal lines are drawn at the mean of each column. Within columns, points are jittered on the *x*-axis for clarity

Next we turn to individual norm differences among Matsigenka and examine the extent to which such differences are predicted by interethnic interaction experience. Figure 3 plots the locations of all 161 interviewees on the latent (meta-norm) axis. As expected, there is marked separation by ethnicity, with Matsigenka tending toward the positive pole for practical interdependence and Mestizos tending toward the negative pole for respectful autonomy. However, it is apparent that a number of Matsigenka individuals have axis locations very similar to those of average Mestizos, suggesting that they hold the Mestizo-typical meta-norm for respectful autonomy. The right three columns of Fig. 3 demonstrate that these Matsigenka with the Mestizo-typical norm tend to have interethnic commerce, wage labor, and education experience. In contrast, Matsigenka with the more Matsigenka-typical norm for practical interdependence may have interethnic commerce and wage labor experience, but almost none have education experience with Mestizos.

**Fig. 4:**
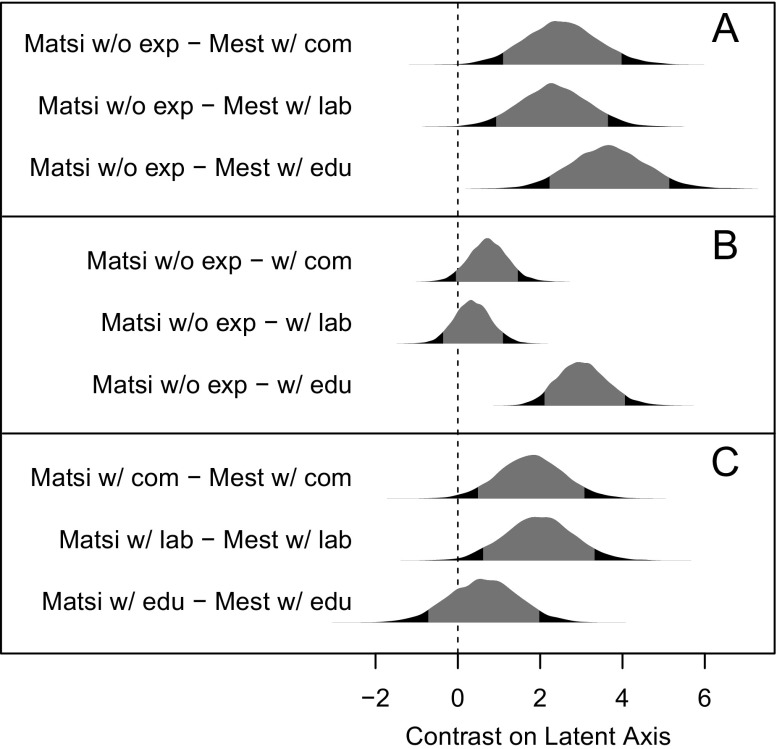
Predicted contrasts on the latent axis for groups defined by ethnicity and interaction experiences with Mestizos, based on the best-fitting IRT model (m19 in ESM Table B1). 90% highest probability density intervals (HPDI) are shown in grey. *Panel A*: Predicted difference in location between an average Matsigenka without any interethnic interaction experience (Matsi w/o exp) and an average Mestizo with only commerce experience with other Mestizos (Mest w/ com) (posterior mean contrast 2.52, 90% HPDI: [1.09, 3.98]), an average Matsigenka without interethnic experience and an average Mestizo with only labor experience with other Mestizos (Mest w/ lab) (2.34, [0.93, 3.65]), and an average Matsigenka without interethnic experience and an average Mestizo with only education experience with other Mestizos (Mest w/ edu) (3.66, [2.22, 5.14]). Here and below, probability distributions that do not strongly overlap zero indicate a detectable difference in location, i.e., in meta-norm. *Panel B*: Predicted difference in location between an average Matsigenka without and with only interethnic commerce (0.74, [−0.05, 1.46]), or wage labor (0.36, [−0.36, 1.09]), or education (3.04, [2.1, 4.06]) experience. *Panel C*: Predicted difference in location between an average Matsigenka with only interethnic commerce, wage labor, or education experience, and an average Mestizo with each type of experience only among other Mestizos (respectively, 1.78, [0.49, 3.08]; 1.98, [0.61, 3.33]; 0.62, [−0.73, 1.99])

The same effect is seen in Fig. 4, plotting counterfactual contrasts of posterior predictions from the best-fitting model (m19 in ESM Table B1, with 33% of model weight). The model predicts that Matsigenka who have never engaged in commerce, wage labor, or education interactions with Mestizos have the Matsigenka-typical meta-norm of practical interdependence, which differs from the Mestizo-typical meta-norm of respectful autonomy (Fig. 4a). Similarly, Matsigenka with commerce and wage labor experience tend to hold the norm of practical interdependence like those of other Matsigenka (Fig. 4b), but unlike the norm of most Mestizos (Fig. 4c). In contrast, Matsigenka who went to school with Mestizos tend to hold the norm of respectful autonomy like most Mestizos (Fig. 4c), and unlike the norm typical of their fellow Matsigenka (Fig. 4b) (further details of analysis in ESM B).

An exploratory model including sex and categorical age predictors achieved 7% of model weight, although the coefficients of each of these predictors could not be distinguished from zero (m12 in ESM Table B1). Furthermore, coefficient estimates for ethnicity and the interethnic experience predictors were not distinguishable from those of the best-fitting model, above. This gives us confidence that inclusion of sex and age in the model has little effect on the results. Coefficient estimates for all models are provided in ESM Table B1.

### Bargaining

There is ethnographic evidence that Matsigenka have less bargaining power in the domain of interethnic education than they do in interethnic wage labor and commerce. This coincides with the result above that Matsigenka with interethnic education experience are more likely to hold Mestizo-typical norms than are Matsigenka with interethnic labor and commerce experience. In the context of a strict boarding school environment, Matsigenka children usually do not control their presence or absence in classrooms, nor the time they must allocate to studying, which constitute their contributions to coordination in the domain of education. Additionally, students usually cannot choose their coordination partners—in other words, the Mestizo teachers and caretakers with whom they interact. By leveraging their physical strength and institutional authority to adjust punishments and incentives, teachers can thus compel students to coordinate using Mestizo norms, which many students likely adopt and internalize. Mestizo-run secondary schools are attractive to many Matsigenka parents and children, and they generally function near maximum capacity. Thus, competition among Mestizo-run schools for Matsigenka students is largely absent.

In contrast, for both wage labor and commerce, Matsigenka control valued resources for coordination: their labor and their money, respectively. Because laborers and customers are usually in demand, Mestizos compete among themselves to interact with Matsigenka, who can often choose to work for or buy from those Mestizos most willing to coordinate using Matsigenka-typical norms. For instance, Matsigenka wage laborers usually have the option of changing employers if Mestizos try to impose Mestizo-typical norms either inside or outside of the context of labor. As a Mestizo from Atalaya who routinely contracted Matsigenka field hands stated,

[Matsigenka] work well, but I let them work in their own way, because their world is different. A type of person like us [Mestizos] already knows how this kind of work is. [We] work until late, [and are] more demanding. But a Machi, when you bring him [to the agricultural field], you let him work in his own way. If he wants to leave, then we leave. If he wants to go for a little while because he is tired, I let him . . . If I say something to them, like demanding that they do something, they get angry and they leave you, just like that. They’re not even interested in the money.

Thus, relative to education, there may be less incentive for Matsigenka to adopt Mestizo-typical norms during interaction in domains such as wage labor and commerce, where Matsigenka have greater bargaining power (additional examples in ESM C.2).

### Interaction-Frequency-Biased Norm Adoption

It seems likely that Matsigenka directly adopt Mestizo-typical norms as a result of interethnic coordination interactions. For instance, describing Matsigenka boarding school students in Boca Manu, who also engage extensively in commerce and wage labor on weekends, one Mestizo resident stated,

When they first come, they have many problems, and they are used to not working. That is one of their customs. And when they come here they have to change their way of thinking. They have to see how to get ahead. It’s a struggle for them. He who does the most, is the most ambitious, obtains the best things. They see that among themselves, and they change.

There is evidence of a positive relationship between the frequency of interethnic interaction and the probability of holding out-group norms. Relative to Matsigenka with interethnic wage labor and commerce experience, Matsigenka who went to school with Mestizos tend to have the most Mestizo-like norms and also the most lifetime interethnic interaction experience. For instance, of the 16 Matsigenka who attended Mestizo-run schools, 14 also have interethnic wage labor experience, which is often quite extensive. Thus, Matsigenka with interethnic education experience tend to have more years of interaction experience with Mestizos in the combined domains of education and labor (~12 years, on average) than do Matsigenka wage laborers who did not attend a Mestizo-run school (~ 4 years, on average). It is thus likely that the total amount of interaction experience with Mestizos, regardless of domain, is positively associated with Mestizo-like norms among Matsigenka.

However, it is also likely that education has a unique effect on out-group norm adoption, independent of the frequency or amount of time spent in interaction. Two Matsigenka interviewees did not go to Mestizo-run schools but have approximately 15 years of wage labor experience, living and working for more than half of each year with Mestizos outside of Tayakome. Despite this extensive interethnic experience, these individuals tend to have more Matsigenka-typical norms (larger α_*j*_) than do the nine Matsigenka who each have combined education and labor experience with Mestizos totaling approximately 10 to 15 years (ESM Figure C1). This anecdotal evidence suggests that, on average, accounting for the total amount of interethnic experience, interethnic education is more strongly associated with Mestizo-like norms than is wage labor alone.

### Interethnic Assortment on Norms

There is ethnographic evidence for a moderate degree of assortment on norms in interethnic interaction. Matsigenka children with the most Mestizo-like norms may be preferentially selected for, and retained in, Mestizo boarding schools. For instance, two of the three Mestizo boarding secondary schools currently attended by Tayakome children require matriculation interviews, including a cursory evaluation of norms (e.g., readiness to shake hands and return a greeting in Spanish). Additionally, several Matsigenka students have been expelled from these two schools for failure to conform to Mestizo norms of acceptable students (e.g., obedient and nulliparous). In contrast, one of the three boarding schools attended by Tayakome students does not require matriculation interviews and has not, to our knowledge, expelled students. Thus, at this school, initial assortment on norms is not enforced from the Mestizo side of the interethnic interaction. Similarly, in informal conversations in Tayakome, several Matsigenka with very Matsigenka-typical norms (high α_*j*_) expressed a strong desire, and detailed future plans, to engage in wage labor interactions with Mestizos (ESM Figure C1). Thus, assortment on norms may occur in some contexts of interethnic education, but it may be weak or absent for many other Matsigenka-Mestizo interactions.

In order for interethnic assortment on norms to affect norm dynamics, the norms of Matsigenka with Mestizo-typical norms should be preferentially copied by their fellow Matsigenka (i.e., success- or prestige-biased intra-ethnic social learning). Although we did not explicitly investigate such a preference in the present study, we note its plausibility. For example, the mother of a Mestizo-educated Matsigenka man in Tayakome told JAB that she stopped wearing a traditional Matsigenka nose ring (*koriki*) at the insistence of her son, whom she esteems, and who had apparently adopted a Mestizo norm for the inappropriateness of such ornaments.

### Success-Biased Interethnic Social Learning

As shown above, the probability of holding Mestizo-typical norms is contingent on the type of interethnic interaction experience of Matsigenka individuals (i.e., commerce or labor versus education). This is contrary to the expectation under success-biased social learning, assuming Matsigenka regard Mestizos as successful or prestigious, and assuming all Matsigenka regardless of interethnic experience are aware of Mestizo-typical norms. Although Mestizos do control access to desired manufactured goods, JAB’s ethnographic observations suggest that most Matsigenka may not generally regard the Mestizos of Boca Manu and Atalaya as more prestigious than they are. For instance, a Matsigenka with extensive experience working on a Mestizo-owned tourist boat in Atalaya viewed Mestizos as inferior workers, stating,

Mestizos are a little lazy. Matsigenka surpass them in work. As crew members, sometimes [the boss] tells us to wash the boat. The Mestizo doesn’t wash; he just stays watching. The Matsigenka washes everything.

If interethnic differences in prestige or perceived success are small, success-biased social learning will be weak. On the other hand, it may also be the case that many of the norms represented by the vignette questions are difficult for Matsigenka to observe, or hear about, without extensive experience living among Mestizos. However, once a Matsigenka learns of such norms, she may adopt them via success-biased social learning even if she never employs such norms in her personal interactions with Mestizos. There is some limited evidence for this type of learning. Among the twenty Matsigenka with four or more years of combined interethnic education and wage labor experience, approximately half held a Mestizo-typical (respectful autonomy) norm concerning inheritance (Question 7, 11/20 interviewees), compared with only 15% (9/59) among Matsigenka with less-extensive interethnic experience. This occurred despite the fact that only four Matsigenka interviewees have had the opportunity to coordinate with Mestizos in the context of inheritance (two have children with a Mestizo, and two have one Mestizo parent). Thus, success-biased social learning may occur to a limited extent among Matsigenka with extensive interethnic interaction experience.

### Childhood Socialization

There is an association between early age of interethnic socialization and the probability of holding out-group-typical norms. Matsigenka interviewees who engaged in interethnic interaction at the earliest ages were those who attended Mestizo-run schools. These included five students who attended just interethnic primary school (when aged approximately 6–12), five who just attended interethnic secondary school (when aged approximately 13–17) or beyond, and six who attended both interethnic primary and secondary schools. As shown above, for Matsigenka, interethnic education has the strongest association with Mestizo-typical norms. Socialization of children to a broad range of Mestizo norms, including those outside of academics, appears to be one goal of Mestizo-run education. For instance, a Mestizo staff member at one boarding school attended by Tayakome children explained:

In the boarding school there are rules. . . . [What] we’re doing here is simply the way to put away dishes, [to] put things in order, [to go about] cleaning, [to] have a clean room, [to] have everything in order, [to] live with beauty and honor.

No Matsigenka engaged in interethnic wage labor or commerce as a young child without also attending Mestizo schools. Thus, in this sample, the effect of early age of out-group socialization on norm adoption cannot be distinguished from that of other aspects of interethnic education experience.

## Discussion

Our objective was to investigate the mechanisms by which individuals adopt out-group norms, potentially leading to cultural dynamics in ethnically structured populations. We have shown quantitatively that indigenous Matsigenka who attended Mestizo-run schools tend to hold more Mestizo-typical norms across a range of social contexts, relative to Matsigenka with only wage labor and commerce experience with Mestizos. Cross-sectional data of this type cannot be used directly as evidence that interethnic interaction, such as education, causes cultural change (see also Graves 1967). However, we have leveraged our ethnographic observations to show that the observed pattern is likely due to all five of the mechanisms we identified as potentially influencing individual adoption of out-group cultural norms: bargaining, interaction-frequency-biased norm adoption, interethnic assortment on norms, success-biased interethnic social learning, and childhood socialization. Below we argue that, of these mechanisms, bargaining is particularly important in this ethnographic context and may also explain why interethnic education can be such a potent driver of cultural dynamics in other ethnic groups engaging with colonial powers. However, first we address limitations and strengths of our methodology and analysis.

### Limitations and Strengths of the Study

This study systematically examined norms, but not the behaviors to which they apply. Norms and behavior may correspond (e.g., Atran et al. 2002), though they need not. Of particular importance are cases of bicultural competence (LaFromboise et al. 1993), in which an individual may modify her behavior according to the norms of the ethnic group in which she finds herself. In this study we assume that each individual holds a single norm with respect to each vignette question (or a single meta-norm applicable to each question), and that this norm is reflected in each response. Under this assumption, if the behavior of a biculturally competent individual changes with ethnic context, it may therefore periodically deviate from the norms that she personally holds. It is possible that, rather than reflecting individually held norms, vignette responses of a biculturally competent individual reflect behavioral decisions corresponding to the ethnic context that she imagines while listening to each vignette (which are mostly designed to be neutral with regard to Matsigenka and Mestizo ethnic context). This study is limited by the assumption that any such imagined context is one in which the subjectively appropriate behavior corresponds to the norms that the interviewee personally holds.

We employed Bayesian item-response models to analyze individual-level responses to a range of questions about norms. Although similar in underlying mathematical form to the widely used cultural consensus model framework (Romney et al. 1986), IRT models are more flexible, and, most importantly, facilitate direct estimation of the effects of predictors on latent outcome variables in hierarchically structured data (Schacht and Grote 2015; Oravecz et al. 2014). An important goal of this study is to assess between-group variation and explain within-group variation in a variety of norms, apparent in Fig. 2. The IRT models allow us to represent this norm variation in a lower-dimensional space, where effects of the hypothesized predictor variables can be meaningfully estimated and understood. We expect this analytical strategy to prove particularly useful for anthropological interview data in any context where the researcher employs a battery of questions to investigate and explain individual-level variation in cognitive states (e.g., norms, preferences, beliefs) that cannot be directly observed. We encourage other researchers to explore our analysis scripts (https://github.com/jabunce/bunce-mcelreath-HN-2016-matsigenka-norms) and modify them to suit their needs.

### Bargaining Power Interacts with Other Mechanisms

The low bargaining power of Matsigenka children in Mestizo-run schools interacts with other identified mechanisms of norm adoption. For instance, the fact that Mestizo-educated Matsigenka have more lifetime interethnic interactions than Matsigenka without such experience may contribute to their adoption of Mestizo-typical norms over the course of their lives. However, it also seems likely that Mestizo-typical norms learned in school facilitate subsequent interethnic coordination in domains such as commerce and wage labor. After graduating, Mestizo-educated Matsigenka may self-assort into commerce and wage-labor coordination interactions with Mestizos, domains in which subsequent norm adoption may occur, though at comparatively lower rates owing to the increased bargaining power of Matsigenka. Thus, adoption of Mestizo-typical norms by Matsigenka students with low bargaining power may facilitate subsequent interaction-frequency-biased norm adoption.

The low bargaining power of Matsigenka students may cause feelings of inferiority. In a boarding school environment, Matsigenka children may view Mestizo teachers and peers as more prestigious and successful, thereby facilitating success-biased social learning of Mestizo norms unrelated to those that Matsigenka actually use to interact with Mestizos. In contrast, in domains such as wage labor, the higher bargaining power of Matsigenka may reduce feelings of inferiority and thereby reduce the success-biased adoption of such norms. Thus, bargaining power may interact with success-biased interethnic social learning.

Finally, the young age of Matsigenka students may contribute to their susceptibility to adoption of Mestizo-typical norms. However, consistent with JAB’s observations, Ochs and Izquierdo (2009) argue that Matsigenka children as young as six years old have already learned important Matsigenka-typical norms, such as sharing, hard work, and intragroup harmony. Simple exposure to alternative Mestizo-typical norms would not necessarily induce these children to change the norms they have already learned. Thus, in addition to early exposure, the potent effect of interethnic education on norms is likely also due to the fact that children are easier to physically manage and strategically isolate from elder co-ethnics (e.g., in a boarding school), such that their control over coordination resources and choice of adult coordination partners is constrained, thereby reducing bargaining power. In summary, the low bargaining power of Matsigenka students likely plays an important role in norm adoption, despite the fact that other identified mechanisms may operate concurrently.

### Education, Bargaining Power, and Cultural Change

The potency of education as a driver of cultural change has long been recognized and exploited by colonial powers attempting to eradicate the cultural norms of indigenous peoples (e.g., to “develop” or “civilize” them), including Native South Americans (Aikman 2003), Native North Americans (Parliament of Canada 1985; Trennert 1979), and Indians under British colonial rule (Seth 2007). The contemporary movement for intercultural bilingual education attempts to combat this legacy. One strategy is to introduce non-Western students to desired Western academic knowledge using the pedagogical norms of the students’ own society, and to require local community oversight of, and participation in, students’ education (Aikman 2003; Trapnell 2003). Only local communities themselves can judge the appropriateness of such an educational program. However, we note that several elements of this approach may, in effect, increase the relative bargaining power of indigenous students and parents in educational coordination with out-group teachers: for example, community choice of teachers (hiring and firing power), students learn from both co-ethnics and out-group members, attendance is voluntary. Thus, if such a program is effective in transcending the historical paradigm of colonial education by reducing the loss of valued ethnic-typical cultural norms, an increase in relative bargaining power may be an important part of the explanation.

We argue that differential bargaining power is likely to be one powerful driver of cultural dynamics in contexts of interethnic interaction, and it is at least partially responsible for the pattern among Matsigenka that out-group cultural norms strongly co-vary with interethnic education experience. Bargaining power can vary by domain of interethnic interaction (e.g., education versus wage labor), affecting the costs and benefits accruing to individuals of such interactions. Consequently, even when norm distributions differ across an ethnic boundary, as they do here, interethnic interaction does not invariably entail miscoordination costs for all participants (contra McElreath et al. 2003), and it does not always lead to the erosion of ethnic-typical cultural variation. Similarly, although ethnic communities may have compelling reasons to curtail it, the adoption of out-group norms, when it does occur, should not be viewed as invariably costly. Rather, to advance theory of cultural change in ethnically structured populations, interethnic norm adoption decisions can be more productively investigated as resulting from the interaction of several mechanisms, including strategic choice of benefit-seeking individuals in contexts of low bargaining power. Future work in this and other ethnographic settings should incorporate longitudinal data on individual norm development and look for situations affording natural experimental control over interethnic interaction experience, in order to more effectively distinguish among the predictions of the five mechanisms of norm adoption identified here.

## Electronic supplementary material

ESM 1 (PDF 616 kb)
