# Supplementary material for: Interethnic Interaction, Strategic Bargaining Power, and the Dynamics of Cultural Norms: A Field Study in an Amazonian Population
Source: Hum Nat. 2017 Aug 18;28(4):434–56. doi: 10.1007/s12110-017-9297-8 (PMC5662675; doi:10.1007/s12110-017-9297-8)
Supplement: Supplementary file 1 — (PDF 616 kb) [file 12110_2017_9297_MOESM1_ESM.pdf]

Electronic Supplemental Material (ESM)

Interethnic Interaction, Strategic Bargaining Power, and the Dynamics of Cultural Norms: A Field Study in an Amazonian Population

Human Nature 28(4), 2017, http://dx.doi.org/10.1007/s12110-017-9297-8

John Andrew Bunce[1,2,3] and Richard McElreath[1,2]

[1]Department of Human Behavior, Ecology, and Culture, Max Planck Institute for Evolutionary Anthropology, Leipzig, Germany
[2]Department of Anthropology, University of California, Davis, CA USA
[3]Department of Anthropology, Indiana University, Bloomington, IN USA

Corresponding author: John Andrew Bunce (john_bunce@eva.mpg.de)

A. Supplemental Methods

A.1. Study Population

Matsigenka Study Population

This study was conducted among residents of the Matsigenka Native Community of Tayakome, located inside Manu National Park, in the Department of Madre de Dios, in the lowland Amazonian region of Southeastern Peru. The Matsigenka (also spelled Machiguenga or Matsiguenka) are indigenous Amazonians who speak a language in the Arawakan family. Most currently live in the watersheds of the Urubamba and Manu Rivers in the Departments of Cusco and Madre de Dios (respectively) in lower-elevation tropical forests. The current population is estimated at 12,000 across both watersheds (Shepard 2002). Dialects, subsistence strategies, market integration, and certain other aspects of culture differ to varying degrees between Urubamba and Manu, with Manu populations (the focus of this study) generally less-well integrated into the local market economy.

Tayakome, with a population of 79 adults (plus one health care technician in the health post) in 41 nuclear families, in 12 household clusters (or clans) at the time of this study, is the smaller of two officially recognized Matsigenka Native Communities inside Manu National Park, a UNESCO Biosphere Reserve and World Heritage Site. The community was established in the late 1960s by missionaries of the Summer Institute of Linguistics, which were evicted from Tayakome after creation of the national park in 1972 (Shepard et al. 2010). As a consequence of living inside a national Protected Natural Area, residents are required to maintain "traditional" subsistence practices of swidden horticulture (mostly manioc), fishing, and bow hunting, which are believed to be less detrimental to the extraordinarily high biodiversity of the park (Levi et al. 2009; Ohl-Schacherer et al. 2007). Domesticated animals (excepting dogs and chickens), firearms, and the extraction and sale of forest products are prohibited by the park administration. These restrictions have been, and continue to be, a source of tension between the Matsigenka and park authorities (Shepard et al. 2010). Tayakome can only be reached by a one- to two-day boat trip up the Manu River from the nearest Mestizo town of Boca Manu, located

just outside the park boundary. Non-Matsigenka may only enter the park and visit Tayakome while conducting government-sanctioned activities (e.g., medical services, Catholic religious services, research and infrastructure projects, monitoring by park guards). There is a government health post in Tayakome staffed by one or two Mestizo technicians, as well as a primary school staffed by two externally-trained Matsigenka teachers. Nearly all Matsigenka in Tayakome prefer to communicate exclusively in their native language. However, most adults can speak and understand basic Spanish, and a few are fluently bilingual. At the time of this study, there were three battery-powered short-wave radios in Tayakome, and a satellite pay telephone at the health post (seldom used by residents). There were no functioning televisions. However three households had devices capable of playing dvd movies. Some households had battery-powered stereos to play recorded music. An NGO had recently installed an ingenious potable running water system (http://www.rainforestflow.org/index.html), and the regional government had recently provided each household with a solar panel, battery, and two light-bulbs. All cooking was done over fire.

Most inter-ethnic interaction between Matsigenka and majority-culture Mestizos occurs outside the park in the domains of boarding school education, wage labor, and commerce. Upon completion of primary school (usually 12 to 15 years old), 15 Tayakome children have been sent to four-year boarding high schools in the Mestizo towns of Boca Manu, Shintuya, and Salvación, all within a one- to two-day boat trip from Tayakome. At the time of this study, 4 of these young adults had successfully completed high school. Additionally, 5 current adult residents of Tayakome grew up outside of the park (e.g., in Urubamba) and attended primary and/or secondary schools in other Mestizo towns. Approximately half of Tayakome adults have varying degrees of experience working as wage-laborers among Mestizos outside of the park. This experience can include harvesting Mestizo banana fields over the course of a few weeks, or working as a crewmember in a Mestizo logging outfit or on a Mestizo-owned tourist boat for months at a time. Most adult Tayakome residents have experience buying goods in Mestizo general stores in towns like Boca Manu, using money earned through wage labor. Commonly purchased items include clothes, flashlights, batteries, soap, machetes, pots, crackers, and carbonated drinks. It is also common for Matsigenka arriving without money in a Mestizo town to initially exchange labor for food and lodging. A Catholic priest visits Tayakome twice per year to hold mass, baptize children, and distribute gifts in well-attended ceremonies.

Mestizo Study Population

This study was also conducted among residents of the Mestizo towns of Boca Manu and Atalaya, located just outside the boundary of Manu National Park, in the Departments of Madre de Dios and Cusco, respectively. Travel down the Alto Madre de Dios River from Atalaya to Boca Manu takes approximately one day. Most Mestizos living in the towns bordering the park are colonists (or their descendants) originating from highland regions of Puno, Cusco, and Apurimac. Many are native Quechua speakers, all are fluent in Spanish, and nearly all prefer to interact among themselves exclusively in Spanish. As a convenience, we use the term Mestizo to refer to the non-Matsigenka residents of these towns. However, it must be noted that few of these individuals actually use this term to refer to themselves. When asked to distinguish themselves from the Matsigenka, colonists from the highlands often referred to themselves as “Gente Blanca” (White People), a label with complex meanings that do not, in this case, correspond objectively with skin color. Additionally, there are residents of Mestizo towns in the region who identify as members of other local indigenous groups, such as Yine and Harakmbut, or as having

mixed indigenous-colonist ethnicity. These individuals make up a small minority in the present study, and, as they are all well-integrated into the market economy, are grouped in the Mestizo category.

Boca Manu is a Mestizo town with a population of approximately 80 adults in approximately 55 family units at the time of this study. The population of this town fluctuates, as many families spend months away from town working in other areas. There are also several residents who are unmarried, or whose spouses live elsewhere. It is located at the entrance to Manu National Park, at the confluence of the Manu and Alto Madre de Dios Rivers, and can only be reached by boat. It is the seat of government for the District of Fitzcarrald, and the Matsigenka communities inside Manu National Park compose an important constituency for the mayor in Boca Manu. Most residents of Boca Manu are Mestizos, although there are three Matsigenka families that live in the town. Residents tend small general stores (five in town), own small restaurants (four in town), make traditional wooden boats (mostly to sell downriver to small-scale gold miners), buy permits to log on nearby indigenous territory, and/or capture and sell valuable tree-trunks that float down the Manu River and out of the park in the rainy season (October – March). Few residents have agricultural fields, and most buy at least some of their food at the general stores, whose merchandise is periodically restocked from Cusco. There is a kindergarten, primary, and secondary school in Boca Manu, including a boarding house close to the school for Matsigenka secondary-school students from the Manu communities. There is a government health post with a resident doctor and technician in town. A generator provides central electricity for three hours each night. Most families have a television, and most people have cellular telephones. A public computer with internet access in the municipal government building is used infrequently. Most Boca Manu residents have interacted at some point with adult Matsigenka from Tayakome, either selling them food or merchandise, or hiring them as part of logging crews. A few men from Boca Manu have visited Tayakome, either as part of political campaigns during mayoral elections, or as boat crews on government-sanctioned business. Nearly all residents are familiar with (know the names of), have sold to, served meals to, or hired as day-laborers, the Matsigenka students in the boarding school. Most residents of Boca Manu identify as belonging to either Catholic, Evangelical, or Adventist congregations in town.

Atalaya is a Mestizo town with a population of approximately 65 adults in approximately 30 households at the time of this study. The population of this town fluctuates due to seasonal workers. It is located on the Alto Madre de Dios River and is the first river terminus of the only road (unpaved) connecting this region to the highland city of Cusco. It is the primary river embarkation point for tourists traveling down from Cusco who have booked boat tours of Manu National Park. Residents tend small general stores (three in town), own small restaurants (six in town), and/or own or work as crewmembers on boats taking tourists for several-day cruises into Manu National Park. Several residents have agricultural fields in which they grow plantains for market in Cusco, a day's truck ride away. Almost no one in Atalaya grows food for auto-consumption. The larger town of Pilcopata is one hour by truck or motorcycle from Atalaya, and most residents buy food in the market there, though smaller items may be purchased in the local general stores. There is a kindergarten and primary school in Atalaya, and secondary (high) schools in the nearby towns of Pilcopata and Salvación. The nearest boarding school housing Matsigenka students is in Salvación (an hour away by truck), and most Atalaya residents have little contact with Matsigenka children. While there is no health post in Atalaya, there are small hospitals nearby in Pilcopata and Salvación. However, many residents prefer to go to Cusco for moderate to serious healthcare needs. Central electricity is available continuously. Most families

have a television, and most people have cellular telephones. Several fee-for-use computers with internet access are used occasionally. At the time of this study, there was one resident Matsigenka family in Atalaya, and two unmarried Matsigenka young adults living with, and working for, a Mestizo family. Several Matsigenka men from Tayakome are well-known to most residents as they spend up to seven months each year living in Atalaya and working as crewmembers on tourist boats. When they don't have tours scheduled, these men are often hired out as day-laborers in Mestizo plantain fields. A few Mestizo men from Atalaya have visited Tayakome as boat crews on government-sanctioned business. Most residents identify as Catholic, Evangelical, or Adventist, but no religious services are conducted in town.

A.2. Data Collection

In Tayakome and Atalaya, JAB lived with a host family, routinely participating in household and community activities and sharing meals. In Boca Manu, he lived in a rented room, ate at local restaurants, and participated in activities such as boat building and wood extraction at the invitation of residents. In all three communities JAB conducted several rounds of individual semi-structured and structured interviews with adult residents. All interviews were conducted by him in the interviewee's preferred language (Matsigenka or Spanish) while out of earshot of other community members. He conducted at least one interview with all but two residents of Tayakome, and with approximately two thirds of the residents of Boca Manu and Atalaya. Included in this study are interviews with members of two of the three Matsigenka families living in Boca Manu, two Matsigenka residents of Atalaya, as well as one Mestizo technician working in the health post in Tayakome. The first rounds of interviews entailed soliciting a life-history narrative. From these interviews, JAB identified education, labor and commerce as three domains of common Matsigenka-Mestizo inter-ethnic interaction. In follow-up interviews he recorded each individual's recollection of personal intra- and inter-ethnic experience in these three domains. Based on his own experience living in these communities, he then designed a set of fourteen vignette questions (Bernard 2006), presented below. From the perspective of this study, none of the vignette questions has a correct or incorrect answer, so no value is assigned to affirmative versus negative responses to any question. In cases of individuals who are cross-culturally competent, we assume that these vignette questions measure an individual's favored norms. Thus, during some inter-ethnic interactions, such an individual may actually coordinate using non-favored norms which do not coincide with her answers to the vignette questions (e.g., Mestizos hiring Matsigenka wage laborers).

Below are the fourteen vignette questions employed in this study, across eight social coordination contexts. JAB developed questions in Spanish with the help of an English-Spanish bilingual Peruvian collaborator. He translated the questions into Matsigenka, and then had them back-translated from Matsigenka to Spanish with the help of a Matsigenka-Spanish bilingual Matsigenka collaborator in Tayakome. Due to organizational difficulties, questions were presented in the same order to interviewees from Tayakome and Boca Manu, but the order of questions 13 and 14 changed for interviewees in Atalaya.

Interviewees responded affirmatively or negatively after JAB initially presented each vignette question. For a subset of interviewees, he then asked them why they responded the way they did. Upon hearing his request for an explanation, occasionally they then changed their answer from negative to affirmative, or vice versa. If the interviewee did not wish to explain why they changed their initial answer, we did not include their response to that particular question in the present analysis. We exclude these responses in case JAB's request to explain

their initial answer led them to believe that he was not in agreement with their answer, which they then changed in order to answer in the way they believed he wanted them to. This occurred despite assurances that their answers were confidential and that JAB believed there were no right or wrong answers to the vignette questions. These cases of response changes may be due to the fact that interviewees were unaccustomed to answering such unusual questions in this unfamiliar format. On other occasions, some interviewees explained that their response (affirmative or negative) to a vignette question was contingent on additional information that JAB had not provided in the vignette itself. For example, for Question 1 (below), an informant responded that it is okay for a wife to go hunting sometimes while her husband stays home, but it is not okay if she does it often. Such responses resulted from interviewees thinking more deeply about a vignette than JAB had when he designed it. We excluded these contingent responses from the present analysis, because JAB had not provided the requested details (e.g., frequency of the behavior in question) to the rest of the interviewees. For all those interviewees who answered the question without requesting additional details, we assume that they interpreted the question in a similar way (e.g., the wife always goes hunting, not just sometimes). As a result of these two classes of interviewer-interviewee misunderstanding, we excluded a total 73 out of 2094 responses (3.5%). To check the effect of excluding these data, we included people's initial responses, and included contingent responses after providing the additional requested information such that these interviewees interpreted the questions in the way we assumed all other interviewees had. Including or excluding these data has no qualitative effect on the conclusions.

The latent axis constructed by the domain-general IRT analysis from these fourteen vignette questions is interpreted according to how individuals with different positions on the latent axis answered each question. The negative pole of the axis corresponds to a meta-norm prioritizing respectful autonomy in interaction behavior. "Respectful" here refers to formality, honor, and conforming to the stereotype of a "civilized" member of Peruvian national society. Autonomy is here defined as individualism and self-reliance. The positive pole of the latent axis corresponds to a meta-norm emphasizing practical interdependence. "Practical" here stands for prioritizing immediate material benefits over conformity to custom or societal expectations, e.g., valuing money over honor. Interdependence here stands for altruistic behavior (e.g., obedience) benefiting the social group, which, in this case, is usually the family unit or, at its largest, the clan (of which there are 12 in Tayakome). Explanations of how answers to each vignette question contribute to interpretation of the latent axis are provided below each translation. Note that there are multiple ways of interpreting the answers to each question. We provide a set of interpretations that emphasize one common theme uniting most of the questions. This theme constitutes one meaning of the latent axis constructed through the IRT analysis. There are undoubtedly other themes uniting the questions, which would lead to alternative, and equally correct, interpretations of the latent axis. Note, however, that interpretation of the meaning of the latent axis has no effect on the conclusions of this study. The latent axis is important only insofar as it distinguishes Matsigenka and Mestizos on the basis of norms, whatever those norms may be. The contribution of each question to the construction of the latent axis through the IRT models is shown in Figure B1, below.

The social context of each vignette question (education, wage labor, commerce, spousal relations, parent-offspring relations, inheritance, healthcare, and religion) is included in parentheses.

Question 1, wife hunts/works (Spousal Relations)

There is a married couple with no children. The woman hunts and fishes (Mestizo: has job and makes more money). The man stays home and cooks, weaves (Mestizo: cleans), and washes clothes. Is this okay or not okay?

Hay una pareja sin hijos. La mujer trabaja y gana plata. El hombre se queda en la casa, cocina, limpia, y lava ropa. ¿Está bien?

Ainho ogari tsinane, mameri otomi, mameri oshinto. Iroro ogotake omatsigatake ochacopi cameti. Onti kovinsari. Iroro oati onkogira ivatsa inkenishkue. Yogari ojina ainho iripitake ivanko, yonkotia sekatsi, intiri ijamatia, ikiviasarati igamisate. Cameti o tera cameti?

Interpretation for latent axis: An affirmative response suggests a more flexible and practical subsistence strategy, allowing the family unit to adapt to a wider range of ecological, economic, and social conditions, thus prioritizing well-being of the family group over individual conformity to gender roles, e.g., those stereotypical of "civilized" Peruvian society. Differences between questions asked of Matsigenka and Mestizo interviewees reflect the different tasks stereotypical of each gender in the two ethnic groups.

Question 2, daughter babysits (Parent-Offspring Relations)

After school, a ten-year-old daughter cannot go to friend's house to play because she has to care for her two-year-old brother until their parents come home at night. Is this okay?

Después de salir de la escuela en la tarde, una hija de diez años no puede ir a jugar con sus amigas porque tiene que volver a la casa y cuidar a su hermano menor de dos años hasta que lleguen sus papás en la noche. ¿Está bien?

Oga oshinto oshiriagakota diez años. Hatanai chavini, okonteiganae escuela. Hanki agavea omagempitira. Ogari oatae ovankokue ogierira iariri itiomiani ishiriagakota dos años, iripigaira iriegi tsitenigeti. Cameti o tera cameti?

Interpretation for latent axis: An affirmative response suggests prioritizing a child's responsibility to the family group over her individual desire to play.

Question 3, not wear dead hat (Inheritance)

A man always wears his favorite hat. After he dies, his son takes the hat and wears it. When he wears it he remembers his father. Is this okay?

Un hombre siempre lleva un gorro favorito. Se muere. El hijo coge el gorro y se lo pone. Cuando se lo pone, piensa en su papá. ¿Está bien?

Yogari sidadi kantani yamea igorate. Imbogini ikamake. Itomi yagakero igorate irashi iriri ikamake. Itomi kantani igoraterora, kantani ikenkirira iriri. Cameti o tera cameti?

Interpretation for latent axis: A negative response corresponds with a common Matsigenka belief that the soul of a dead person will return to haunt anyone who appropriates his or her old possessions, making them sick (J. Bunce, pers. obs., see also Johnson 2003). To prevent this, used (but not new unused) belongings of a dead person should be burned or buried. Because of the physical proximity and spiritual bonds between the bodies of Matsigenka family members, sickness poses a threat to the whole family (Izquierdo et al. 2008). Thus a negative response to this vignette suggests prioritizing wellbeing of the family-group over an individual desire to remember a loved one. This cultural practice also, incidentally, curtails most inter-generational transmission of what little material wealth individual Matsigenka accumulate in their lifetimes.

Question 4, hit students (Education)

A teacher hits students when they don't learn. Is this okay?

Un profesor golpea a sus alumnos cuando no aprenden. ¿Está bien?

Yogotagantasirira ipasatakeri estudiante tera irogoigia sankevantera. Cameti o tera cameti?

Interpretation for latent axis: An affirmative response suggests prioritizing obedience and extrinsic motivation for learning over strategies to stimulate individual student interest and to accommodate variation in individual learning style.

Question 5, no questions (Education)

A student pays attention to the teacher and never asks any questions. Is this okay?

Un estudiante siempre escucha y hace caso al profesor. Nunca hace ninguna pregunta. ¿Está bien?

Yogari estudiante kantani ikemisantake yogatagantasirira. Tenka inkogakote. Cameti o tera cameti?

Interpretation for latent axis: An affirmative response suggests prioritizing conformity and faithful copying of information over curiosity and exploration.

Question 6, post flu (Healthcare)

If you get a respiratory illness (influenza), do you first go to the health post, first use home remedies, or first go to a shaman or curandero? (Affirmative: go first to the health post. Negative: anything else)

¿Si tiene gripe, Ud. va primero a la posta, primero usa remedios caseros, o primero va al curandero?

Agakempi merentsi. Okityo piatake postakue? Okityo povetsike inchashi pankotsikue? Okityo piatake irishipokempira? Okityo piatake iritasonkempira?

Interpretation for latent axis: An affirmative response for the Matsigenka may suggest that, as respiratory infections originate almost exclusively from outside the community and are viewed as non-Matsigenka diseases, they must be treated with non-Matsigenka remedies at the health post. Additionally, as respiratory infections are particularly virulent among the Matsigenka and usually spread rapidly through the community, prompt individual treatment benefits the community. A negative response for Mestizos (nearly always indicating home remedies) suggests that they recognize the inefficacy of most medicines (e.g., antibiotics) for treatment of minor respiratory infections, which are usually viral. It may also suggest a norm for self-reliance, rather than dependence on doctors.

Question 7, pot to needy (Inheritance)

An old woman has two new pots and two adult daughters. One daughter has her own two pots, but wants her mother's pots. The other daughter has no pots, and also wants her mother's pots. When the mother dies, who should inherit the pots? (Illustrated with a diagram. Affirmative = one pot to each daughter. Negative = both pots to the daughter who has none)

Hay una mujer vieja con dos ollas nuevas. Tiene dos hijas adultas. Una hija tiene sus propias dos ollas, pero quiere las ollas de su mama. La otra hija no tiene ollas. También quiere las ollas de su mama. ¿Cuando la mama se muere, a quién debería heredar las dos ollas?

Ogari tsinane okamake. Aityo pitieti ojiromanga otierira. Ainho piteni oshinto antaroni. Paniro oshinto aityo pitieti ohiromangane. Okogake oka otierira hiromanga. Ogari apiteni oshinto mameri ohiromangane. Ariompa okogake oka otierira hiromanga. Tyani gakerone otierira hiromanga ashi iniro?

Interpretation for latent axis: An affirmative response suggests prioritizing individual entitlement over the needs of others in the kin group.

Question 8, good nonbaptized no heaven (Religion)

A good person does not want to be baptized. Where does his or her soul go when they die? Options: up (heaven), down (hell), somewhere else. (Affirmative: heaven. Negative: hell or somewhere else)

Una persona es buena, pero no quiere bautizarse. ¿Cuando se muere, adónde va su alma?

Yogari sidadi inti cameti. Tenka ikogira ibautizatakeri Padre. Ikamake. Tyara iriatae isire? Enokue? Saviakue? Pashini?

Interpretation for latent axis: An affirmative response suggests a belief that individual autonomous actions are more important in determining supernatural benefits than is formal acceptance by a representative of an organized religious community. For some Matsigenka, there is a distinction between a soul going into the ground (kipatsikue) and going to a place with flames below the world (morekakue) (see also Rosengren 2004). Both of these places can be indicated by the preposition "down" (saviakue), but they are not necessarily equivalent in terms

of favorable or unfavorable outcomes after death. JAB realized this after most of the interviews were completed. Therefore, a negative response to this question by Matsigenka may be interpreted as "anywhere other than a heaven-like place above the world".

Question 9, bad baptized heaven (Religion)

A bad person is baptized. Where does her or his soul go when they die? Options: up (heaven), down (hell), somewhere else. (Affirmative: heaven. Negative: hell or somewhere else)

Una persona es mala gente. Es bautizado. ¿Cuando se muere, adónde va su alma?

Yogari sidadi inti tera cameti. Padre ibautizatakeri. Ikamake. Tyara iriatae isire?

Interpretation for latent axis: An affirmative response suggests a belief that once a person is socially accepted as a Christian, he or she will receive the supernatural reward due Christians, regardless of autonomous individual behavior. Negative responses by Matsigenka should be interpreted as they are for Question 11, above.

Question 10, stop work to visit (Wage Labor)

A man is hired to prepare an agricultural field. He stops work at noon in order to go visit a friend. He returns the next day to finish the job. Is this okay?

Un hombre está contratado limpiar una chacra. Deja de trabajar a mediodía porque quiere visitar a su amigo. El día siguiente termina de limpiar la chacra. ¿Está bien?

Kamatitya, ipuinatakeri koriki yogari sidadi itsamaitera imagashipogote irashi Viracocha. Katinga poriatsiri, yoga sidadi yapakuianae itsamaitera, iroventi ikogake ikamosote ishaninka. Paita onkuita yagatake itsamaitera, itsongatero imagashipogote irashi Viracocha. Cameti o tera cameti?

Interpretation for latent axis: An affirmative response suggests prioritizing the maintenance of social bonds over dedication to contractual labor.

Question 11, cheap mean store (Commerce)

There are two stores. One is cheap with a mean owner. The other is expensive with a nice owner. Where would you buy? (Affirmative: expensive store)

Hay dos tiendas. Una tienda es barata pero el dueño es malo. La otra tienda es cara pero el dueño es bueno. ¿Dónde iría Ud. para comprar?

Aityo pitieti otiendate Viracocha. Patiro otiendate tenka opuinatasanotempa. Yogari shintarorira inti kisantari. Apiteni otiendate onti puinatasanori. Yoga shintarorira tenka irikisante. Tyaka picompratake? Hanta puinatasanori irashi cameti shintarorira o apiteni tenka opuinatasanotempa irashi kisantari?

Interpretation for latent axis: An affirmative response suggests prioritizing respectful behavior over the practicality of saving money.

Question 12, xcousin marriage (Spousal Relations)

A man wants to marry. His mother is the sister of the woman's father. Is it okay for him to marry this woman? (Illustrated with a diagram. For the Matsigenka, examples were provided of potential marriages between people known to the interviewee.)

Un hombre quiere casarse con una mujer. La madre de él es la hermana del padre de la mujer. ¿Está bien?

Yogari sidadi ikogake ijinantempera. Iriniro onti iritsiro iri oga tsinane. Cameti o tera cameti?

Interpretation for latent axis: An affirmative response indicates acceptance of cross-cousin marriage, which may serve to strengthen social ties within clans. This suggests prioritizing benefits to the clan (e.g., cohesion) over any perceived or un-perceived individual-level benefits associated with avoidance of consanguineous mating. Note that an affirmative response does not necessarily indicate a preference for cross-cousin marriage over other forms of marriage. Rather it indicates a lack of opposition to cross-cousin marriage.

Question 13, arranged marriage (Parent-Offspring Relations)

Parents want their daughter to marry a certain man that she does not like. She wants to marry someone else. Should she obey her parents and marry him anyway or not? (Affirmative = she marries who she wants and does not obey her parents)

Los papás quieren que su hija se case con un chico. Pero a la hija no le gusta el chico. Ella quiere otro. ¿Debería hacerles caso a sus papás y casarse con ese chico, o no?

Iriegi ikogake ojinantempera irishinto antaroni. Ikantakero "Noshinto, gaeri yogari sidadi, inti cameti." Kantangicha, ishinto tenka okogiri yoga sidadi, okogakeri pashini. Iroventi, iroro, irishinto, tenka okematsataeri iri. Tenka ojinantaigiri yoga sidadi. Agakeri pashini. Cameti o tera cameti?

Interpretation for latent axis: An affirmative response suggests prioritizing individual choice over obedience to the family group.

Question 14, laborer party (Wage Labor)

A man is hired to work two days: Monday and Tuesday. Monday night there is a party (Matsigenka: hosted by a Matsigenka). Should he go and get drunk? (Matsigenka: He goes and gets so drunk that he can’t work on Tuesday. Is this ok?) (Affirmative: it is okay to go and get drunk. Negative: it is not okay to go and/or not okay get drunk)

Un hombre está contratado trabajar dos días: lunes y martes. Lunes en la noche hay una fiesta. ¿El hombre debería ir y emborracharse?

Itinkame ipuinatakeri koriki yogari sidadi intsamaitera pitieti kuitagita: Lunes y Martes. Lunes tsitenigeti aityo owidoki irashi ishaninka. Iriro iati, para ishinkitaka. Tenka iragavea intsamaitira Martes. Cameti o tera cameti?

Interpretation for latent axis: A positive response suggests prioritizing the maintenance of social (i.e., friendship and kin) bonds over honoring an individual labor contract. The differences between questions asked of Matsigenka and Mestizo interviewees result from the fact that contractual labor occurs exclusively in Mestizo towns. A party there would nearly always be hosted by a resident Mestizo, and a Matsigenka might not be invited or feel comfortable going. Thus, for Matsigenka interviewees JAB specified that the party was hosted by a Matsigenka. He also specified a more stringent condition for Matsigenka interviewees, namely, that the laborer is so drunk that he cannot work the next day. Even with this difference in stringency, the only interviewees who answered positively were Matsigenka. It should be noted that inebriation is an important part of both Mestizo and Matsigenka society. Among Mestizos it usually involves mass-produced beer, is generally viewed as a vice, and is both cause and consequence of much domestic discord. In contrast, among Matsigenka, women control production and serving of a fermented manioc drink called owidoki (Spanish: masato). Owidoki parties usually take place at a couple's home and are important social events attended by many community members. Both men and women drink, and often become drunk. Gossip, myths, songs, and (often ingeniously irreverent) jokes are shared, as well as grievances with other community members. Such parties are one of the important contexts in which it is socially acceptable to air (and often resolve) such grievances (Shepard 2002), as well as transmit important cultural knowledge such as myths and songs. The importance of these parties to Matsigenka culture may be the reason why only Matsigenka believed it acceptable to attend such a party at the expense of a contractual labor obligation.

A.3. Statistical Analysis

A.3.1. General Description

Based on the life history interviews, JAB developed hypotheses (*a priori* relative to model construction) that four binary predictors would be important in determining an individual's norms, and hence her location on the latent axis: Ethnicity (1=Matsigenka, 0=Mestizo), Education (1=school with Mestizos, 0=no such experience), Labor (1=wage labor with Mestizos, 0=no such experience), and Commerce (1=commerce or exchange with Mestizos, 0=no such experience). For further description of these predictors, see Appendix section A.3.3., below. Note that Matsigenka individuals may take on values of 0 or 1 for Education, Labor, and Commerce predictors, while all Mestizo participants in this sample have values of 1 for these three predictors. We did not have *a priori* hypotheses about the effects of sex or age on norms, but we included these in the models as exploratory predictors. We also included the following two-way interactions: Ethnicity and Education, Ethnicity and Labor, Ethnicity and Commerce, and Ethnicity and Sex. Explanations of how these predictors were coded is provided below. Further information and examples of IRT models can be found in Schacht and Grote (2015), Jackman (2001), Fox(2010), and Koster et al. (2016).

Because these data were collected across three communities (Tayakome, Boca Manu, and Atalaya), it is reasonable to include a random effect for community in the IRT models. However, community and ethnicity are highly correlated in this population, as very few Matsigenka live in Atalaya and Boca Manu, and no Mestizos live in Tayakome (with the exception of the health post technician). For most questions, mean responses from Mestizo residents of Boca Manu and Atalaya were very similar. Therefore, the analysis presented here does not include a random effect for community. Including a community-level random effect in the best-fitting model does not qualitatively change the interpretations presented below.

As unidimensional IRT models provide a reasonably good fit to these data (indicated by all discrimination parameters estimated as non-zero: Figure B1, below), and as we had no *a priori* hypotheses of multiple simultaneous meta-norms, here we do not pursue higher-dimensional models. Following Jackman (2001), the justification for using discrimination parameters to decide on the number of dimensions to model in an exploratory analysis is as follows. If, in a one-dimensional model, a given question's discrimination parameter is zero, then an individual's position on the latent axis provides no information about how that individual answered that particular question. If several questions are found to have discrimination parameters of zero, then perhaps the responses of these questions are strongly correlated among themselves, but not detectably correlated with responses to the rest of the questions. This would justify fitting a two-dimensional model, in the hope that the discrimination parameters of these questions will be non-zero in the second dimension. Jackman (2001) provides an example of such data exploration. However, for a model of given dimension, if the discrimination parameters of all questions are estimated to be non-zero, then an individual's position in the modeled latent space is informative with regard to how she answered all questions. This is the case for the one-dimensional models used here.

A series of 19 models (Appendix Table B1, below) with different combinations of predictors for ethnicity, inter-ethnic education, wage labor, and commerce experience with Mestizos, as well as sex, age, and interactions was fit to the responses of the fourteen vignette questions (see Appendix A.2., above). Models have the following generic form, but vary in the fixed-effect predictors (e.g., b_1 for Ethnicity) included in the linear function for α_j:

$$\Pr(y_{jk} = 1) = \text{logit}^{-1}[\gamma_k(\alpha_j - \beta_k)]$$

$$\alpha_j = b_0 + b_{indiv[j]} + b_1 x_{1[j]} \ \dots , \qquad \text{for } j = 1, \dots, J$$

$$b_0 \sim \text{N}(0, 1),$$

$$b_{indiv[j]} \sim \text{N}(0, 1), \qquad \text{for } j = 1, \dots, J$$

$$b_1 \sim \text{N}(0, 1),$$

$$\beta_k \sim N(0, \sigma_\beta^2), \qquad \text{for } k = 1, \dots, K$$

$$\gamma_k \sim N\left(0, \sigma_\gamma^2\right), \qquad \text{for } k = 1, \dots, K$$

$$\sigma_\beta^2 \sim \text{Exponential}(1)$$

$$\sigma_\gamma^2 \sim \text{Exponential}(1),$$

where J is the number of interviewees and K is the number of vignette questions. See Bafumi et al. (2005) for notational changes if not all interviewees answered all questions. Informative $\mathrm{N}(0,1)$ priors for the intercept (b_0), random effect for individual (b_{indiv}), and additional predictor coefficients (e.g., b_1) identify the model by constraining the position and scale of the α_j's to fall within a reasonable distance (e.g., usually within two standard deviations) on either side of zero, resolving additive and multiplicative aliasing (Bafumi et al. 2005). Any choice of position and scale in IRT models is arbitrary and results in equivalent inference. Reflection (or rotational) invariance (Jackman 2001; Bafumi et al. 2005) is not a concern as mean discriminations of all questions are positive, effectively polarizing the latent axis. Variances for β and γ are given exponential hyperpriors to control ceiling effects common to logistic models (McElreath 2016, pg 378). The complete series of 19 models is shown in Table B1, below. Parameter estimation for each model was accomplished with RStan (Stan Development Team 2016), running four Hamiltonian Monte Carlo chains in parallel until convergence was indicated by a high effective number of samples (> 500) and $\hat{R}$ estimates of 1.00 (McElreath 2016). This usually entailed 4000 samples per chain, half of which were warm-up. Data and analysis script are available from Github (https://github.com/jabunce/bunce-mcelreath-HN-2016-matsigenka-norms).

A.3.2. Coding of Fixed-Effect Predictors

Ethnicity (1=Matsigenka)

Interviewees were coded as Matsigenka if they self-identified as Matsigenka. Two siblings had a Matsigenka mother and a Mestizo father. Both of them had lived for several years in Matsigenka communities in Manu, self-identified as Matsigenka, and were coded as such. All non-Matsigenka in this study were classified as Mestizos. A description of these interviewees is provided above in Appendix A.1.

Sex (1=Male)

Sex was coded according to self-identified gender.

Age Category (Adolescent, Adult, Elder)

Many Matsigenka, especially older adults, do not know their exact age in years. For this reason we use age categories as predictors, estimating ages where necessary. Adolescents were younger than 20, adults were 20 to 50, and elders were older than 50.

Education Experience (1=attended school with Mestizos)

All Mestizos attended primary and/or secondary school with other Mestizos, so all were coded as 1. Several Matsigenka interviewees grew up outside of Tayakome and went to either a boarding- or non-boarding primary school with Mestizos. These individuals were coded as 1. Most Matsigenka in Tayakome attended primary school in Tayakome, with Matsigenka teachers and all Matsigenka students. If this was an interviewee's only education experience, she or he was coded as 0. There is no secondary school in Tayakome. A few Matsigenka from Tayakome attended boarding secondary schools with Mestizos outside of Tayakome for at least four of the requisite five years, and some had additional educational training after high school (e.g., for tour guide certification). These boarding school attendees were coded as 1. Two Matsigenka interviewees attended a boarding secondary school for a few months before either being expelled

or leaving because they did not like it. These interviewees were coded as 0. The average amount of inter-ethnic education experience among Matsigenka scored as 1 was approximately 7 years.

Wage Labor Experience (1=wage labor experience with Mestizos)

All Mestizos were coded as having wage labor experience with other Mestizos. The vast majority of these interviewees, both men and women, had earned money themselves, helped their spouse or relative earn money, and/or hired people to work for them. In contrast to the wage labor experience of most Matsigenka, most Mestizos were self-employed (e.g., banana farmers, small restaurant owners, boat builders) and did not have a "boss". Matsigenka were coded as 1, i.e., having wage labor experience with Mestizos, if they spent an approximate total of at least 12 months living with and working under Mestizos in their lifetime. This experience occurred in Mestizo towns, Mestizo-run tourist lodges or work camps (e.g., for logging or oil companies), or Mestizo-staffed park guard posts. Several Matsigenka interviewees attended boarding secondary school in Mestizo towns for several years and were hired as wage laborers on most weekends. These individuals were also scored as 1. The average amount of wage labor experience among Matsigenka scored as 1 was approximately 5.5 years.

Commerce Experience (1=commerce experience with Mestizos)

All Mestizos were coded as 1, i.e., having commerce experience with other Mestizos, as all had purchased items in stores. Matsigenka were coded as 1 if they had ever directly bought or sold/traded items to Mestizos. Most Matsigenka had bought from Mestizo stores at least a few times in their life, although quantification based on memory was difficult.

A.3.3. Model Comparison and Inference

Models with different combinations of predictors were compared using the Widely Applicable Information Criterion (WAIC) (McElreath 2016, pg 205). Inference was based on the posterior predictive distributions of the best fitting model (Appendix Table B1, below). We then use these posterior predictions to calculate counterfactual contrasts for the position on the latent axis of each combination of ethnicity and interaction experience in order to directly address the hypotheses under investigation (Schenker and Gentleman 2001). The general form of these contrasts is as follows: 1) Matsigenka without experience (i.e., education, labor, or commerce) – Mestizo (all of whom have each type of interaction experience only with other Mestizos); 2) Matsigenka without interaction experience – Matsigenka with each type of inter-ethnic experience; and 3) Matsigenka with inter-ethnic experience – Mestizo with experience only with other Mestizos. Data and analysis scripts used to fit all models using the open-source RStan package (Stan Development Team 2016) in R (R Core Team 2014) are available from Github (https://github.com/jabunce/bunce-mcelreath-HN-2016-matsigenka-norms).

B. Supplemental Results

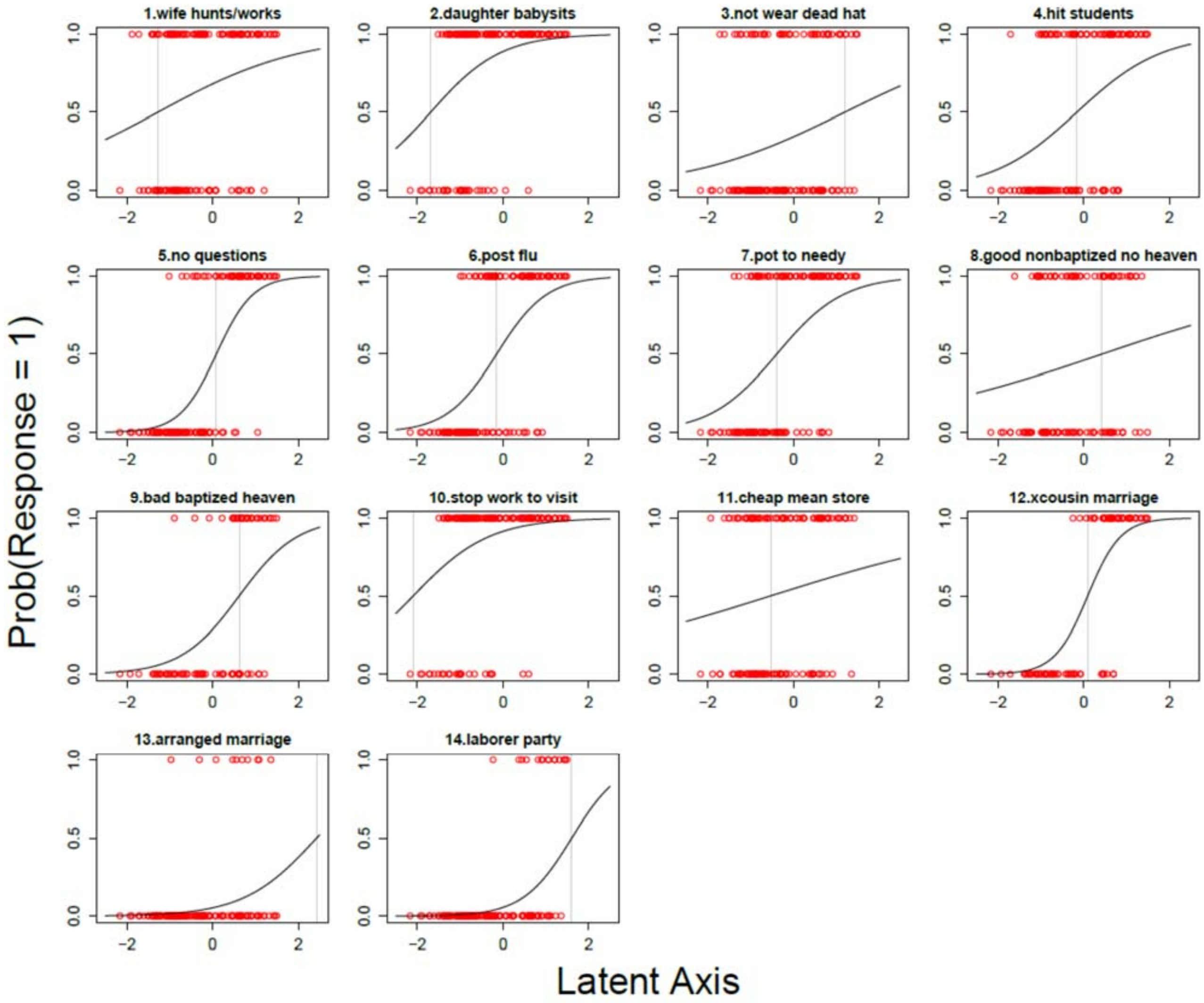

Figure B1. Logistic functions generated from the mean posterior location (β_k, x-value of vertical grey line) and discrimination (γ_k, slope of curve at intersection with vertical line) of each of the 14 vignette questions, derived from an IRT model with a random effect for individual and no fixed effect predictors (m1 in Table B1 below). Questions with higher absolute value discrimination contribute more to the construction (and hence, interpretation) of the latent axis. The discriminations of questions 8 and 11 are marginally distinguishable from zero (Figure B2, below). The fact that discriminations are distinguishable from zero suggests that questions map well onto a single dimension (Jackman 2001). The posterior mean location of each individual on the latent axis is plotted in red. A person's y-value of 0 or 1 corresponds with a respectful autonomy or practical interdependence response, respectively, to the given question. For a given question's location β_k and positive discrimination γ_k, increasing an individual's location α_j increases the probability that she gives the practical interdependence response. For a given α_j and positive γ_k, increasing β_k decreases the probability of a practical interdependence response.

For a given $\alpha_j > \beta_k$, increasing a positive γ_k increases the probability of a practical interdependence response, while simultaneously decreasing the probability of such a response for $\alpha_j < \beta_k$.

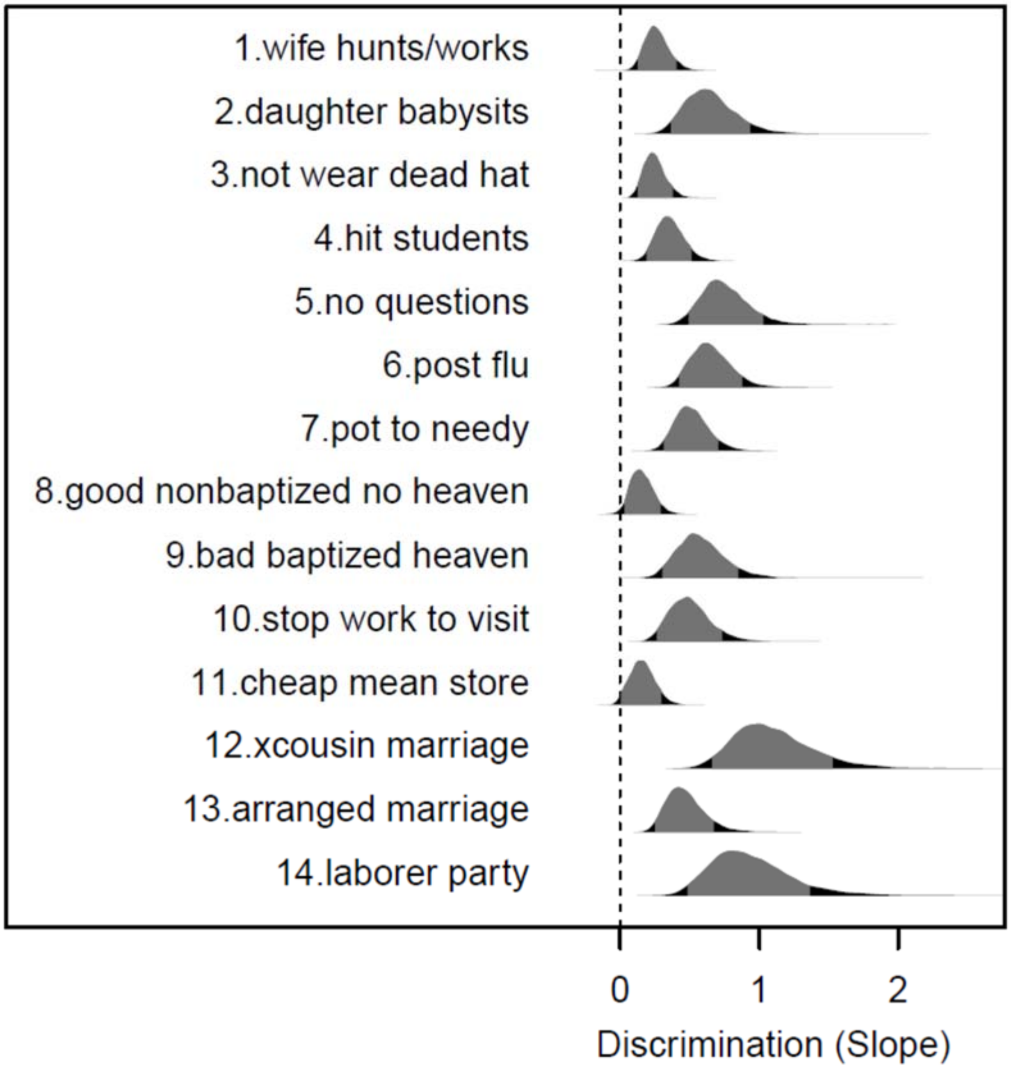

Figure B2. Posterior probability density estimates for discrimination parameters (γ_k) in the best-fitting IRT model (m19 in Table B1) for the fourteen vignette questions. 90% HPDI are shown in grey. Note that all discrimination estimates are markedly non-zero, with the exception of those for questions 8 and 11, which are marginally non-zero. This suggests that nearly all questions contribute substantially to the construction of the latent axis, and supports the decision to limit analysis to a single latent dimension (Jackman 2001).

The best-fitting IRT model (m19 in Table B1, below) contained predictors for ethnicity, education with Mestizos, wage-labor, and commerce experience (and their interactions), but did not include predictors for sex and age category. Strong associations are detected between

TABLE B1. Posterior mean coefficient estimates for IRT models fit in this analysis (with 95% highest probability density interval below each). Models receiving more than 5% WAIC weight are highlighted in grey. The best-fitting model, m19, incorporates all inter-ethnic interaction domains of interest, without the exploratory predictors of sex and age. Column headings are fixed-effect predictors (including interactions), and model weight. Ethn = Ethnicity, Adol = Adolescent, Educ = Education Experience, Labor = Wage Labor Experience, Comm = Commerce Experience.

Model	Ethn	Sex	Ethn X Sex	Adol	Adult	Elder	Educ	Ethn X Educ	Labor	Ethn X Labor	Comm	Ethn X Comm	WAIC weight
m1													1.3e-17
m2	2.59<br>1.96 , 3.26												1.2e-08
m3	2.67<br>1.93 , 3.39	-0.17<br>-0.75 , 0.4	-0.07<br>-0.84 , 0.68										4.3e-09
m4	2.65<br>2.01 , 3.37			-0.27<br>-1.47 , 0.94	-0.42<br>-1.46 , 0.65	-0.32<br>-1.48 , 0.73							5.9e-09
m5	2.73<br>2.01 , 3.54	-0.19<br>-0.79 , 0.42	-0.05<br>-0.83 , 0.74	-0.29<br>-1.47 , 0.9	-0.41<br>-1.45 , 0.67	-0.28<br>-1.43 , 0.76							1.9e-09
m6	1.77<br>0.61 , 3.01	-0.09<br>-0.77 , 0.64	0.22<br>-0.7 , 1.08	-0.05<br>-1.35 , 1.22	0.01<br>-1.13 , 1.12	0.07<br>-1.16 , 1.2	-2.53<br>-3.82 , -1.33	-0.78<br>-2 , 0.49					0.013
m7	1.7<br>0.54 , 2.88	-0.01<br>-0.68 , 0.63	0.41<br>-0.43 , 1.31	-0.11<br>-1.42 , 1.07	0<br>-1.09 , 1.13	-0.13<br>-1.3 , 0.98			-1.76<br>-2.92 , -0.64	0.17<br>-0.98 , 1.33			3e-07
m8	2.14<br>0.95 , 3.36	-0.1<br>-0.74 , 0.5	-0.02<br>-0.84 , 0.79	-0.12<br>-1.35 , 1.08	-0.02<br>-1.12 , 1.08	-0.01<br>-1.18 , 1.12					-1.67<br>-2.81 , -0.49	0.62<br>-0.52 , 1.75	1.5e-08
m9	1.49<br>0.17 , 2.9	0.01<br>-0.73 , 0.73	0.43<br>-0.59 , 1.38	-0.02<br>-1.38 , 1.25	0.15<br>-1.01 , 1.29	0.09<br>-1.12 , 1.26	-2.17<br>-3.48 , -0.79	-0.96<br>-2.25 , 0.37	-1.04<br>-2.27 , 0.25	0.21<br>-1.03 , 1.48			0.029
m10	1.73<br>0.35 , 3.12	-0.02<br>-0.74 , 0.69	0.27<br>-0.67 , 1.24	0.01<br>-1.26 , 1.32	0.24<br>-0.87 , 1.41	0.19<br>-0.95 , 1.38	-2.35<br>-3.69 , -1.04	-0.96<br>-2.26 , 0.35			-1.15<br>-2.45 , 0.16	0.2<br>-1.08 , 1.42	0.07
m11	1.61<br>0.25 , 3	0.03<br>-0.6 , 0.7	0.4<br>-0.5 , 1.29	-0.04<br>-1.24 , 1.24	0.19<br>-0.97 , 1.28	0<br>-1.18 , 1.15			-1.47<br>-2.75 , -0.25	0.03<br>-1.21 , 1.28	-1.15<br>-2.47 , 0.04	0.33<br>-0.84 , 1.54	4.3e-07
m12	1.54<br>0.11 , 2.98	0.05<br>-0.74 , 0.8	0.44<br>-0.57 , 1.46	0<br>-1.34 , 1.24	0.3<br>-0.84 , 1.49	0.16<br>-1.04 , 1.4	-2.1<br>-3.53 , -0.77	-1.06<br>-2.46 , 0.3	-0.87<br>-2.17 , 0.43	0.19<br>-1.14 , 1.51	-0.95<br>-2.29 , 0.4	0.1<br>-1.23 , 1.38	0.07
m13	1.79<br>0.67 , 2.97						-2.47<br>-3.74 , -1.29	-0.71<br>-1.84 , 0.56					0.067
m14	1.79<br>0.67 , 2.9								-1.67<br>-2.75 , -0.56	0.36<br>-0.81 , 1.45			8.9e-07
m15	2.06<br>0.96 , 3.25										-1.67<br>-2.84 , -0.55	0.62<br>-0.51 , 1.68	6.7e-08
m16	1.56<br>0.33 , 2.91						-2.14<br>-3.52 , -0.85	-0.86<br>-2.23 , 0.39	-0.9<br>-2.1 , 0.39	0.35<br>-0.92 , 1.59			0.1
m17	1.79<br>0.44 , 3.08						-2.22<br>-3.51 , -0.9	-0.89<br>-2.17 , 0.46			-1.06<br>-2.33 , 0.17	0.24<br>-0.99 , 1.47	0.32
m18	1.7<br>0.43 , 3								-1.32<br>-2.58 , -0.16	0.17<br>-0.99 , 1.4	-1.11<br>-2.35 , 0.12	0.4<br>-0.84 , 1.58	1.7e-06
m19	1.63<br>0.29 , 3.06						-2.03<br>-3.38 , -0.68	-1.01<br>-2.34 , 0.37	-0.71<br>-2 , 0.56	0.34<br>-0.91 , 1.62	-0.89<br>-2.27 , 0.4	0.15<br>-1.19 , 1.42	0.33

individuals' norms and both ethnicity and education experience, while more modest associations are evident for wage labor and commerce experience, and interactions between ethnicity and experience (Figure B3, below). Sex and age are not present in the best-fitting model, and show no detectable associations with norms represented by the latent axis.

The difference in WAIC between models m19 and m17 is slight, and some sampling iterations give m17 the highest WAIC weight. This suggests that m19 is slightly over-fit, in the sense that, if one knows a person's ethnicity, commerce experience, and education experience, little additional predictive value is gained by also knowing that person's wage labor experience. However, because the objective of this study is to compare the associations between norms and inter-ethnic experience in these three domains, model m19 is used for calculation of contrasts in Figure 4.

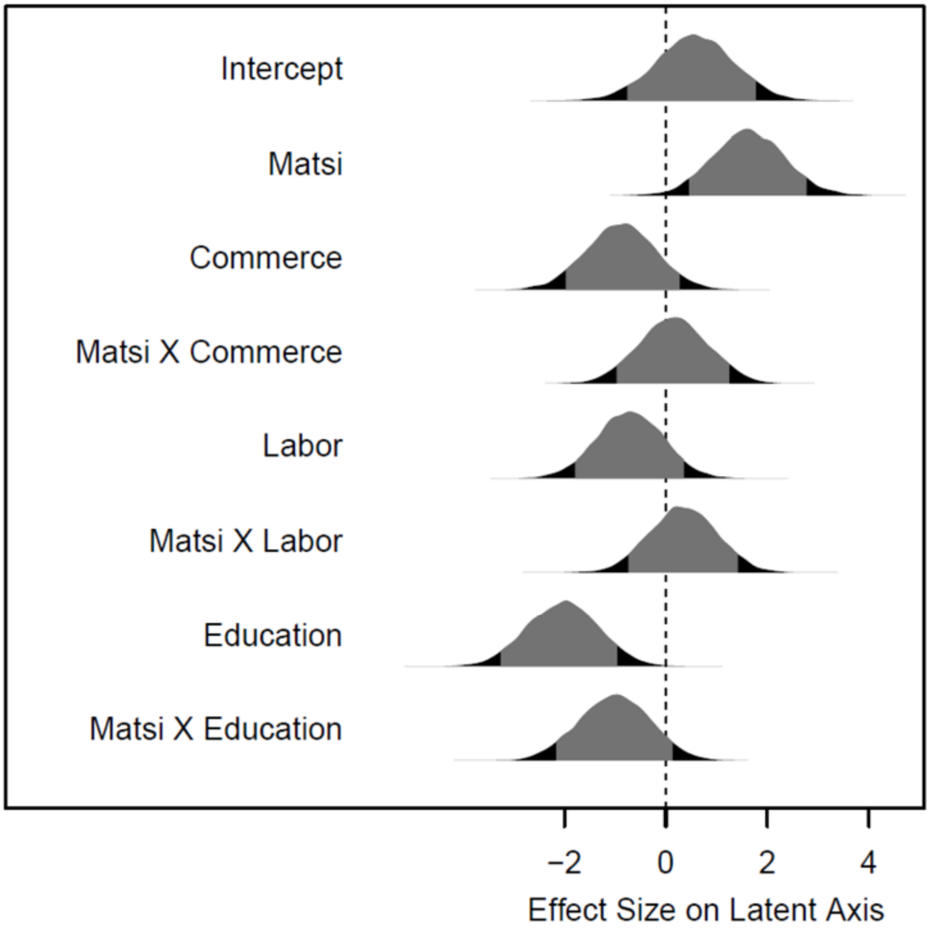

Figure B3. Posterior probability density estimates for parameters in the best-fitting IRT model (m19 in Table B1) fit to all fourteen vignette questions. 90% HPDI are shown in grey. Effect size is on the latent axis, where more-positive values correspond to a domain-general norm for practical interdependence, and more-negative values correspond to a norm for respectful autonomy. Ethnicity (Matsi) and inter-ethnic education experience (Education) have strong associations with individuals' positions on the latent axis, i.e., very little posterior density overlapping zero. Wage labor, commerce, and [ethnicity by experience] interactions have more modest associations.

C. Supplemental Discussion

C.1. Ethnic Differences in Norms

The raw proportions of Matsigenka and Mestizo responses to the vignette questions (Figure 2), as well as IRT analyses (Figures 3 and 4), suggest marked ethnic differences in the distributions of norms across eight contexts of social coordination: commerce, wage labor, education, spousal relations, parent-offspring relations, inheritance, healthcare, and religion. Matsigenka-typical norms, represented by a meta-norm for practical interdependence, coincide with observations of other ethnographers regarding the importance of harmony and cohesion within the Matsigenka extended family (Johnson 2003; Izquierdo et al. 2008; Izquierdo 2005; Ochs and Izquierdo 2009), and that family members, to some extent, share a collective body (Shepard 2002). Interestingly, norms for such social interdependence may not apply to Matsigenka who are not close kin. This coincides with the subsistence autonomy of Matsigenka family units (Johnson 2003), and could explain the individualistic behavior observed by Henrich (2000; 2004) in experimental games that deliberately excluded framing cues of kinship (Hagen and Hammerstein 2006). Mestizo-typical norms, represented by a meta-norm for respectful autonomy, seem apt for pioneers who left kin groups in the highlands to colonize an unfamiliar tropical environment, and who now depend on integration into a competitive local economy. This norm also coincides with JAB's experience living in Mestizo towns, where emphasis is placed on individual self-sufficiency (even among spouses), formality (e.g., using the formal Usted conjugation of Spanish verbs), and adherence to the rituals of "civilized" society (e.g., stylized political speeches at town meetings). Some of these characteristics may stem from processes of ethnic transformation documented among both highland Quechua-speakers (de la Cadena 1992) and lowland mixed-indigenous populations (Gow 1993).

C.2. Matsigenka control of labor resources

Mestizos who attempt to impose Mestizo-typical labor norms on Matsigenka, risk losing their labor. While living in Atalaya, JAB witnessed a tour-boat owner warn one of his Matsigenka bow-men that he wouldn't pay him if he caught him drinking at a local bar. Later, he attempted to convince two of his Matsigenka boat drivers to make a season-long commitment to him and become his permanent employees with healthcare and retirement benefits, while giving him more control over their professional lives. Within a few months, these Matsigenka men had switched to working as temporary employees of other Mestizo boat owners. In Boca Manu, a Mestizo who routinely contracted Matsigenka boarding school boys to help him haul wood on weekends, told me that he had to pay the Matsigenka a day early to be sure that no one else in town would hire them away at the last minute. Thus, in both Atalaya and Boca Manu, Matsigenka workers appear to be in demand, and Mestizos compete for their labor. They nearly always have the option of switching employers if they are dissatisfied with their current employer.

C.3. Amount vs. Type of Inter-Ethnic Interaction Experience

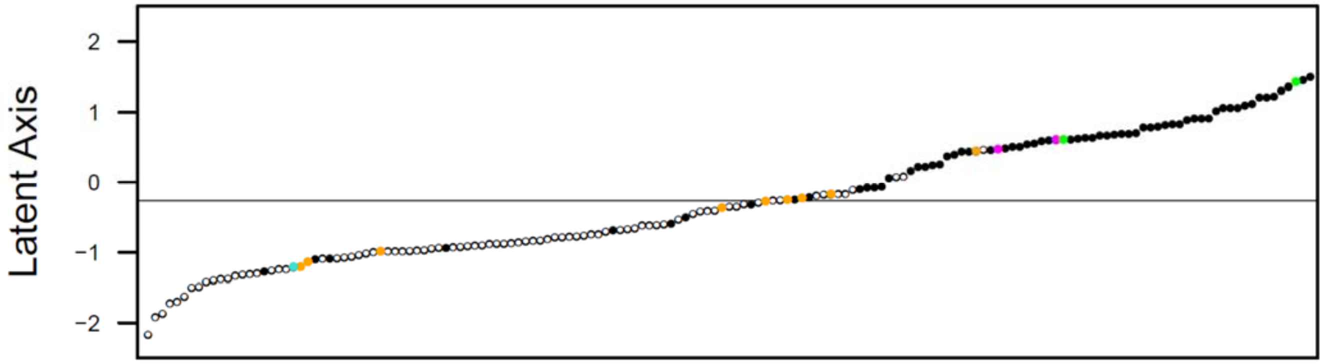

Figure C1. Mean posterior locations of all 161 interviewees on the latent axis, from an IRT model with a random effect for individual and no fixed-effect predictors (m1 in Table B1, above). Mestizos are open circles and Matsigenka are solid circles. The grey horizontal line is the overall population posterior mean location. Purple: two Matsigenka with approximately 15 years of inter-ethnic wage labor experience, and no inter-ethnic education experience. Orange: Matsigenka with a range of approximately 10 to 15 years of combined inter-ethnic education and wage labor experience. Notice that the two purple individuals, with extensive inter-ethnic experience but none in the domain of education, have more-positive values on the latent axis than all orange individuals, who have comparable amounts of inter-ethnic experience including education. This suggests that, for the Matsigenka, after controlling for the total amount of inter-ethnic interaction experience, the domain of inter-ethnic education has a stronger association with the Mestizo-typical meta-norm (more negative values on the latent axis) than does the domain of inter-ethnic wage labor. The blue point in the lower left is an atypical Matsigenka, with a very Mestizo-like meta-norm, who has four years of inter-ethnic wage labor experience and no inter-ethnic education experience. Interestingly, this individual is also unusual for being marginalized by many residents of Tayakome (for reasons withheld to preserve anonymity). Green: two Matsigenka individuals with very Matsigenka-typical norms (high values on the latent axis) who expressed desire and future plans to work as wage laborers with Mestizos. This suggests that these individuals are not concerned (or do not know) that their norms are very different from those of the average Mestizo.

References

Bafumi, J., Gelman, A., Park, D. K., & Kaplan, N. (2005). Practical issues in implementing and understanding Bayesian ideal point estimation. *Political Analysis, 13*(2), 171-187.

Bernard, H. R. (2006). *Research Methods in Anthropology: Qualitative and Quantitative Approaches* (4ed.). Lanham: Altamira.

de la Cadena, M. (1992). Las mujeres son más indias: etnicidad y género en una comunidad del Cuzco. *Isis Internacional, Ediciones de las Mujeres (Santiago de Chile), 16*, 25-71.

Fox, J.-P. (2010). *Bayesian Item Response Modeling: Theory and Applications* (Statistics for Social and Behavioral Sciences). New York: Springer.

Gow, P. (1993). Gringos and wild indians: images of history in western Amazonian cultures. *L'Homme, 33*(126), 327-347.

Hagen, E. H., & Hammerstein, P. (2006). Game theory and human evolution: a critique of some recent interpretations of experimental games. *Theoretical Population Biology, 69*, 339–348.

Henrich, J. (2000). Does culture matter in economic behavior? Ultimatum Game bargaining among the Machiguenga of the Peruvian Amazon. *American Economic Review, 90*(4), 973-979.

Henrich, J., & Smith, N. (2004). Comparative experimental evidence from Machiguenga, Mapuche, Huinca, and American populations. In J. Henrich, R. Boyd, S. Bowles, C. Camerer, E. Fehr, & H. Gintis (Eds.), *Foundations of Human Sociality: Economic Experiments and Ethnographic Evidence from Fifteen Small-Scale Societies* (pp. 125-167). Oxford: Oxford University Press.

Izquierdo, C. (2005). When ''health'' is not enough: societal, individual and biomedical assessments of well-being among the Matsigenka of the Peruvian Amazon. *Social Science and Medicine, 61*, 767-783.

Izquierdo, C., Johnson, A., & Shepard, G. H., Jr. (2008). Revenge, envy, and cultural change in an Amazonian society. In S. Beckerman, & P. Valentine (Eds.), *Revenge in the Cultures of Lowland South America* (pp. 162-186). Gainesville: University of Florida Press.

Jackman, S. (2001). Multidimensional analysis of roll call data via Bayesian simulation: Identification, estimation, inference, and model checking. *Political Analysis, 9*(3), 227-241.

Johnson, A. (2003). *Families of the Forest: The Matsigenka Indians of the Peruvian Amazon*. Berkeley: University of California Press.

Koster, J., Bruno, O., & Burns J. L. (2016) Wisdom of the elders?: ethnobiological knowledge across the lifespan. *Current Anthropology, 57*(1), 113-121.

Levi, T., Shepard, G. H., Jr., Ohl-Schacherer, J., Peres, C. A., & Yu, D. W. (2009). Modelling the long-term sustainability of indigenous hunting in Manu National Park, Peru: landscape-scale management implications for Amazonia. *Journal of Applied Ecology, 46*, 804–814.

McElreath, R. (2016). *Statistical Rethinking: A Bayesian Course with Examples in R and Stan* (Texts in Statistical Science). Boca Raton: CRC Press.

Ochs, E., & Izquierdo, C. (2009). Responsibility in childhood: three developmental trajectories. *Ethos, 37*(4), 391-413.

Ohl-Schacherer, J., Shepard, G. H., Jr., Kaplan, H., Peres, C. A., Levi, T., & Yu, D. W. (2007). The sustainability of subsistence hunting by Matsigenka native communities in Manu National Park, Peru. *Conservation Biology, 21*(5), 1174-1185.

R Core Team (2014). R: a language and environment for statistical computing. R Foundation for Statistical Computing, Vienna, Austria. URL http://www.R-project.org/.

Rosengren, D. (2004). Los Matsigenka. In F. Santos, & F. Barclay (Eds.), *Guía Etnográfica de la Alta Amazonía* (Vol. IV, pp. 1-157, Travaux de l'Institut Français d'Études Andines, Vol. 181). Balboa: Smithsonian Tropical Research Institute, Instituto Francés de Estudios Andinos.

Schacht, R., & Grote, M. (2015). Partner choice decision making and the integration of multiple cues. *Evolution and Human Behavior, 36*, 456-466.

Schenker, N., & Gentleman, J. F. (2001). On judging the significance of differences by examining the overlap between confidence intervals. *The American Statistician, 55*(3), 182-186.

Shepard, G. H. (2002). Three days for weeping: dreams, emotions, and death in the Peruvian Amazon. *Medical Anthropology Quarterly, 16*(2), 200-229.

Shepard, G. H., Rummenhoeller, K., Ohl, J., & Yu, D. W. (2010). Trouble in paradise: indigenous populations, anthropological policies, and biodiversity conservation in Manu National Park, Peru. *Journal of Sustainable Forestry, 29*, 252-301.

Stan Development Team (2016). RStan: the R interface to Stan, Version 2.14.1. http://mc-stan.org.
